# Half of germline pathogenic and likely pathogenic variants found on panel tests do not fulfil NHS testing criteria

**DOI:** 10.1038/s41598-022-06376-4

**Published:** 2022-02-21

**Authors:** Tala Andoni, Jennifer Wiggins, Rachel Robinson, Ruth Charlton, Michael Sandberg, Rosalind Eeles

**Affiliations:** 1 The Institute of Cancer Research, London, UK; 2 Royal Marsden Hospital, London, UK; 3 Leeds Genetics Laboratory, St James’s University Hospital, Leeds, UK; 4 90 Sloane Street, London, UK

**Keywords:** Cancer, Genetics

## Abstract

Genetic testing for cancer predisposition has been curtailed by the cost of sequencing, and testing has been restricted by eligibility criteria. As the cost of sequencing decreases, the question of expanding multi-gene cancer panels to a broader population arises. We evaluated how many additional actionable genetic variants are returned by unrestricted panel testing in the private sector compared to those which would be returned by adhering to current NHS eligibility criteria. We reviewed 152 patients referred for multi-gene cancer panels in the private sector between 2014 and 2016. Genetic counselling and disclosure of all results was standard of care provided by the Consultant. Every panel conducted was compared to current eligibility criteria. A germline pathogenic / likely pathogenic variant (P/LP), in a gene relevant to the personal or family history of cancer, was detected in 15 patients (detection rate of 10%). 46.7% of those found to have the P/LP variants (7 of 15), or 4.6% of the entire set (7 of 152), did not fulfil NHS eligibility criteria. 46.7% of P/LP variants in this study would have been missed by national testing guidelines, all of which were actionable. However, patients who do not fulfil eligibility criteria have a higher Variant of Uncertain Significance (VUS) burden. We demonstrated that the current England NHS threshold for genetic testing is missing pathogenic variants which would alter management in 4.6%, nearly 1 in 20 individuals. However, the clinical service burden that would ensue is a detection of VUS of 34%.

## Introduction

### Background

The landscape of genetic testing for germline cancer predisposition has shifted from single gene testing to multi-gene panels. While the National Health Service (NHS) has integrated some cancer gene panel testing into routine clinical care^1^, there remains debate regarding which of our patients we should be testing. Historically, extensive genetic testing has been curtailed by the cost of DNA sequencing, leading to the establishment of gatekeeping eligibility criteria. With the advent of multiplexed high-throughput sequencing, the cost continues to decline, opening the possibility of testing hereditary cancer predisposition in a wider population^1^, so much so, that some groups have questioned the relevance of genetic testing guidelines altogether^2,3^.

Genetic testing criteria are often based upon a combination of family history, personal demographic and cancer data, and features consistent with cancer syndromes^4^. The National Institute for Health and Care Excellence (NICE) also publishes testing guidelines operating on family history models, and generally sets the cost-effectiveness threshold for gene testing as a carrier probability of 10% or more^5^. NHS England have specified which tests are commissioned by the NHS, and publish criteria-based cancer genetic testing guidelines. Although eligibility criteria for genetic testing assist resource management within the NHS, such criteria remain flawed for the following reasons (Box 1):

**Box 1:** Some problems with current eligibility criteria.

**Challenges of Eligibility Criteria** MISSED: A family history may not be apparent with smaller and blended families UNDERUSED: Non-compliance with standard of care genetic testing, where eligible individuals may not get tested MISUNDERSTOOD: Poor phenotyping of cancer in family histories	

Apparently sporadic cancers may actually be hereditary, missed by family history criteria as modern pedigrees have gradually become smaller and more blended^6^. Although germline *BRCA1* and *BRCA2* (*BRCA1/2*) pathogenic variants account for a large proportion of Hereditary Breast and Ovarian Cancer (HBOC), a Scottish study on *BRCA1/2* testing in ovarian cancer revealed that 48% of pathogenic variants did not fulfil family history criteria for testing^7^. The new Scottish policy of unselected testing of all patients with non-mucinous ovarian cancer increased the annual rate of *BRCA1/2* variant detection five-fold^7^. Similarly, a single-centre study on *RET* gene testing revealed that more than half of patients with a Medullary Thyroid Carcinoma (MTC) and a *RET* pathogenic variant did not have a significant family history of cancer^8^. This discrepancy may also be attributed to de novo cases^9^. Similar studies outside the UK have supported this, with up to 54% of variant carriers being missed due to restrictions of family history criteria^7,10^.

Multiple studies have identified the underuse of genetic testing in cancer patients, despite recommendations^8,11–16^. While testing for *BRCA1/2* variants has gained significant traction^12^, for women with breast cancer meeting eligibility criteria, uptake ranges from 30 to 65%^13^. In American literature, women of African American or Hispanic origin are less likely to be referred to genetics services^12^ and eligibility criteria do not always reflect ancestry-dependent cancer risks^17^. Although it is universally indicated, *BRCA1/2* testing was performed in only 31% of males with breast cancer, and 17.4% of women with ovarian cancer in a study of commercially insured patients in the USA^13^. A study of compliance with testing for Multiple Endocrine Neoplasia Type 2 (MEN2) in patients with MTC revealed 40% did not have *RET* gene testing, which is indicated for all patients with an MTC^8^. A similar story of under-testing and possible under-diagnosis also applies to colorectal cancer^14,15,18^. The misalignment of testing guidelines with clinical practice may be due to a variety of reasons, such as a healthcare professional’s lack of awareness of testing guidelines, resource availability, and population-level behavioural differences leading to patient refusal or non-compliance^12^.

A multi-generational family history is paramount to identification of patients with high-risk cancer susceptibility^19–21^. As genetic testing is integrated into standard practice, a process known as ‘mainstreaming’, the onus is increasingly falling on non-geneticists to take the family history. Prior studies suggest the process of taking a family history can be suboptimal^19,22^, with one study identifying only 61.5% take a family history up to second-degree relatives^11^. Family history criteria often rely on information provided by relatives as electronic records are incomplete and inaccessible across institutions. Situations where difficulties arise include abdominal cancers which are notoriously poorly reported, forms of the disease which are not highly-penetrant^9^, or the patient’s lack of knowledge of their family medical history^23^. Systemic barriers include limited time for the physician or genetic counsellor (GC) to collect a full family history, as well as time-consuming data collection tools^23^.

### The landscape of pathogenic variants

On average, genomic predisposition contributes to 5–10% of all cancers^24–26^, however for certain cancers in certain populations this can be higher (even as high as 30%)^8^. Recognition of this heritability has ever-increasing clinical implications with personalised therapies, as well as ramifications for family cancer surveillance and prophylactic procedures^4^. The spectrum of pathogenic variants associated with cancer ranges from very rare but highly-penetrant, rare moderate-penetrance, to common low-penetrance variants^27^. Rare alleles with higher effect size, such as variants in *BRCA1/2*, are more easily identified^27,28^. For low and moderately penetrant variants, the individual relative risk conferred is smaller, but in combination of multiple variants, the cumulative risk increases^29,30^.

Traditional germline testing has been restricted to high-risk predisposition genes, such as *BRCA1/2*, and mismatch repair (MMR) genes, where classification and management guidelines are better defined. Which genes to include in these panels is determined both at local and regional levels by healthcare institutions and policymakers^31^. Factors influencing this decision include access to funding^1^, known population variants, acceptability to patients, and importantly, the pillars of appropriate test use defined by the ACCE Framework: **A**nalytical validity, **C**linical validity, **C**linical utility, and **E**thical, legal and social implications^31^ (Box 2).

**Box 2:** The ACCE Framework: parameters used to identify appropriate use of genetic testing^31,32^.

**Determining appropriate use of genetic tests** Analytical validity: Is the test accurate at detecting genetic variants? Clinical validity: Is the variant-disease association well-defined, and can we give robust estimates of risk? Clinical utility: Would the result be actionable? Are there effective treatments or prophylactic measures? Ethical, legal and social implications: including obtaining valid consent	

**Figure 1:**
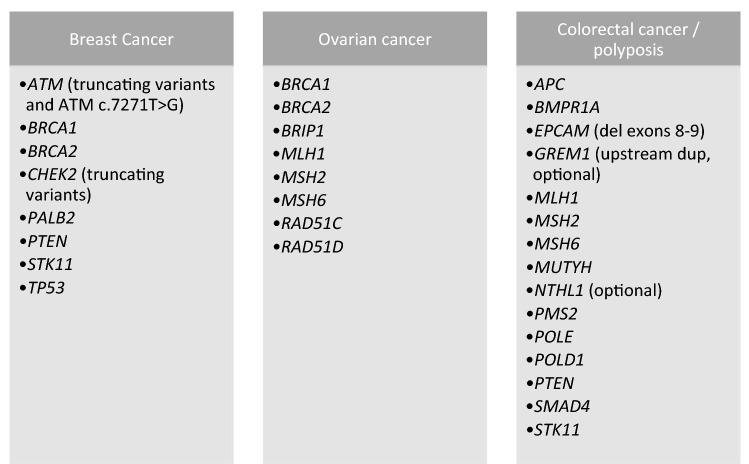
The UK Cancer Genetics Group consensus for genes to be included on cancer panels^1^. Figure created from Taylor et al.^1^.

Tests for an expanding array of high and moderate risk genes are becoming available, but the clinical validity of lower-penetrance or newly-identified variants is open to interpretation^21^. Each test can have its unique implications for patients and their families and there are UK-wide attempts to standardise care nationally^1,33^. The UK Cancer Genetics Group published guidelines on which genes to include in cancer panels based on clinical validity (Fig. 1), and provided management proposals^1^. To assist counselling on clinical validity, predictive computer programs incorporating personal history and family history to calculate risk, such as BOADICEA for breast and ovarian cancer in women, have expanded to include some moderate-risk genes^28^.

The American College of Medical Genetics and Genomics (ACMG) has developed guidelines for more robust variant classification into five categories (Box 3), which relies on multiple factors including population frequency, segregation data, functional and computational modelling, and variant data^34^.

**Box 3:** Classifying sequence variants according to ACMG Guidelines^34^.

**ACMG Classification of Variants** Pathogenic (P) Likely Pathogenic (LP): > 90% certainty of a variant being disease-causing Variant of Uncertain Significance (VUS) Likely Benign: > 90% certainty of a variant being benign Benign	

### Studies that have forgone family history-based testing criteria

Researchers and healthcare professionals have explored the ramifications of simplifying access to testing, and eligibility criteria across a range of populations (Table 1).

**Table 1:** Research studies undertaking genetic testing without the use of family-history based eligibility criteria.

Location	Study	Outcome	Source
Scotland	Testing all women with non-mucinous ovarian cancer for BRCA1/2	Rate of BRCA1/2 variants: 13.1%	^7^
Royal Marsden Hospital, London	Testing all women with non-mucinous ovarian cancer for BRCA1/2	Rate of BRCA1/2 variants: 16%	^35^
University College London, London	Population screening in Ashkenazi Jews compared with family-history based testing	Reduced ovarian and breast cancer incidence by 0.34% and 0.62% respectively, leading to a projected cost reduction of £3.7 million	^36^
Various Institutions, London	Testing BRCA1, BRCA2, RAD51C, RAD51D, BRIP1, PALB2 in an unselected population of women	Population-based testing is more cost-effective than clinical criteria or family-history based testing	^37^
Various Institutions, Australia	Mainstreaming BRCA1/2 testing to all high-grade non-mucinous epithelial ovarian cancer	Rate of detection of BRCA1/2 pathogenic variants of 17%	^38^
Ohio, USA	Gene panel testing in 450 individuals with early onset colorectal cancer	Rate of detection of P/LP of 16% in a wide spectrum of genes (75 genes in 72 people)	^18^
Multi-centre study, USA	Gene panel testing in 959 patients with breast cancer	Overall P/LP rate of 8.65%: 9.39% for patients meeting NCCN testing guidelines, and 7.9% in those who did not	^2^
Multi-centre study, USA	Gene panel testing of all patients presenting with solid tumour cancers, a total of 2984 patients	P/LP rate of 13.3%, VUS rate of 47.4%	^3^

### Our Aim

Our aim was to determine the extent to which more actionable genetic variants are returned by panel testing compared to those that would be returned by criteria-dependent NHS testing of the same genes.

## Methods

### Participants

This study was conducted at a private oncogenetics clinic in London. Patients were included in the study if they underwent cancer predisposition genetic testing between 2014 and 2016. Genetic pre and post-test counselling was provided to all patients as part of standard clinical care. During clinical consultations, discussion of ancestry, particularly Ashkenazi Jewish ancestry, was highlighted as this influenced targeted testing for pathogenic founder variants. Based on the suspected cancer predisposition, specific gene tests were requested for patients. All patients gave their informed consent to have the panel test undertaken by the clinician, and no additional blood samples were taken for this study.

Illumina sequencing was conducted in a UKAS-accredited private laboratory. Variant classification and interpretation were delivered to patients as part of their standard clinical care. The ACGS/ACGM classification was used for this period, prior to the availability of CanVig classification guidelines. Approval for this study was obtained from the Royal Marsden Hospital audit committee. All methods were performed in accordance with hospital guidelines.

### Data collection

Electronic medical records and pedigrees were retrospectively reviewed for information on demographics, and oncological history including age at diagnosis. All patients had an associated family pedigree where data on cancer family history was gathered at the time of the consultation. Box 4 shows information collected on all patients.

**Box 4:** Data collection strategy.

**Data Collection Information** Sex Ancestry Cancer information including type and age at onset (if applicable) Cancer predisposition genes tested Does the patient or their family fulfil panel-specific criteria? Variants detected and classification Were there any unexpected findings? Clinical utility: was there a change in management for the patient or their family?	

Every panel undertaken was compared to the 2020/2021 National Genomic Test Directory Testing Criteria for Rare and Inherited Disease^39^ using the patient information available at the time of the clinical consultation.

### Data analysis

R version 3.5.0 was used for all statistical analysis, and most graphical representations. Microsoft Excel version 16.10 was utilised for the remaining graphics.

## Results

### Patient characteristics

Between 1st January 2014 to 31st December 2016, 152 individuals underwent multi-gene panel testing at an oncogenetics clinic, the majority of whom were of white ancestry (119 patients [78%]). Details on demographics and oncological history are in Table 2.

**Table 2:** Characteristics of study participants.

Characteristic		Number of Patients	% of Patients
Sex	Female	115	75.7
	Male	37	24.3
Race	African	4	2.6
	Arab	18	11.8
	Asian Indian	5	3.3
	Asian Other	2	1.3
	Chinese	4	2.6
	White British	80	52.6
	White European	35	23
	White Other	4	2.6
Ashkenazi Jewish		15	9.9
Personal history of cancer	Affected	98	64.5
	Unaffected	54	35.5

There were 54 unaffected patients (35.5%) who underwent testing, and 98 individuals with single or multiple cancers (64.5%) undergoing diagnostic testing. Of the 98 patients affected, the median *age at cancer onset* was 49 years, ranging from 16 to 82 years of age.

### Clinical characteristics of cancers

The cancer incidence was a total of 104 tumours in 98 patients. The most frequent tumour types were breast cancer (44, [42.3%]), followed by colorectal cancer (24, [23.0%]), prostate cancer (11, [10.6%]), and ovarian cancer (5, [4.8%]).

### Gene panels tested

Based on personal and/or family history of the patient, the clinician selected the relevant panels and genes to be tested. The 3 commonest panels were breast, colorectal, and ovarian cancer panels. Others were familial renal cancer, Fanconi anaemia, familial melanoma, neurofibromatosis, familial pancreatic cancer, Multiple Endocrine Neoplasia (MEN) syndromes, phaeochromocytoma and paraganglioma, retinoblastoma, and *DICER1* gene testing (‘Pleuropulmonary blastoma/Goitre, multinodular 1, with or without Sertoli-Leydig cell tumours’). Supplementary Table 1 outlines the genes tested for each patient.

### Gene panel findings

**Figure 2:**
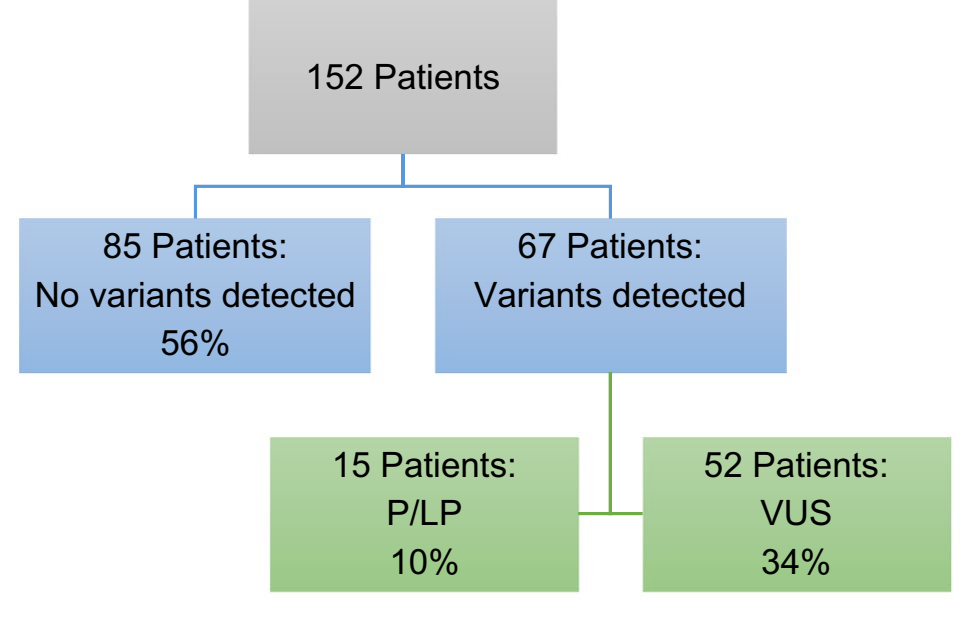
Overall results of gene panel testing. Of note, the VUS rate does not include VUS detected in patients with P/LP variants.

Gene panel sequencing yielded variants in 67 individuals. No variant was detected in 85 individuals (56%), while 34% of people had at least one VUS, and 10% had variants which were P/LP (Fig. 2).

### Cancer genes implicated

**Figure 3:**
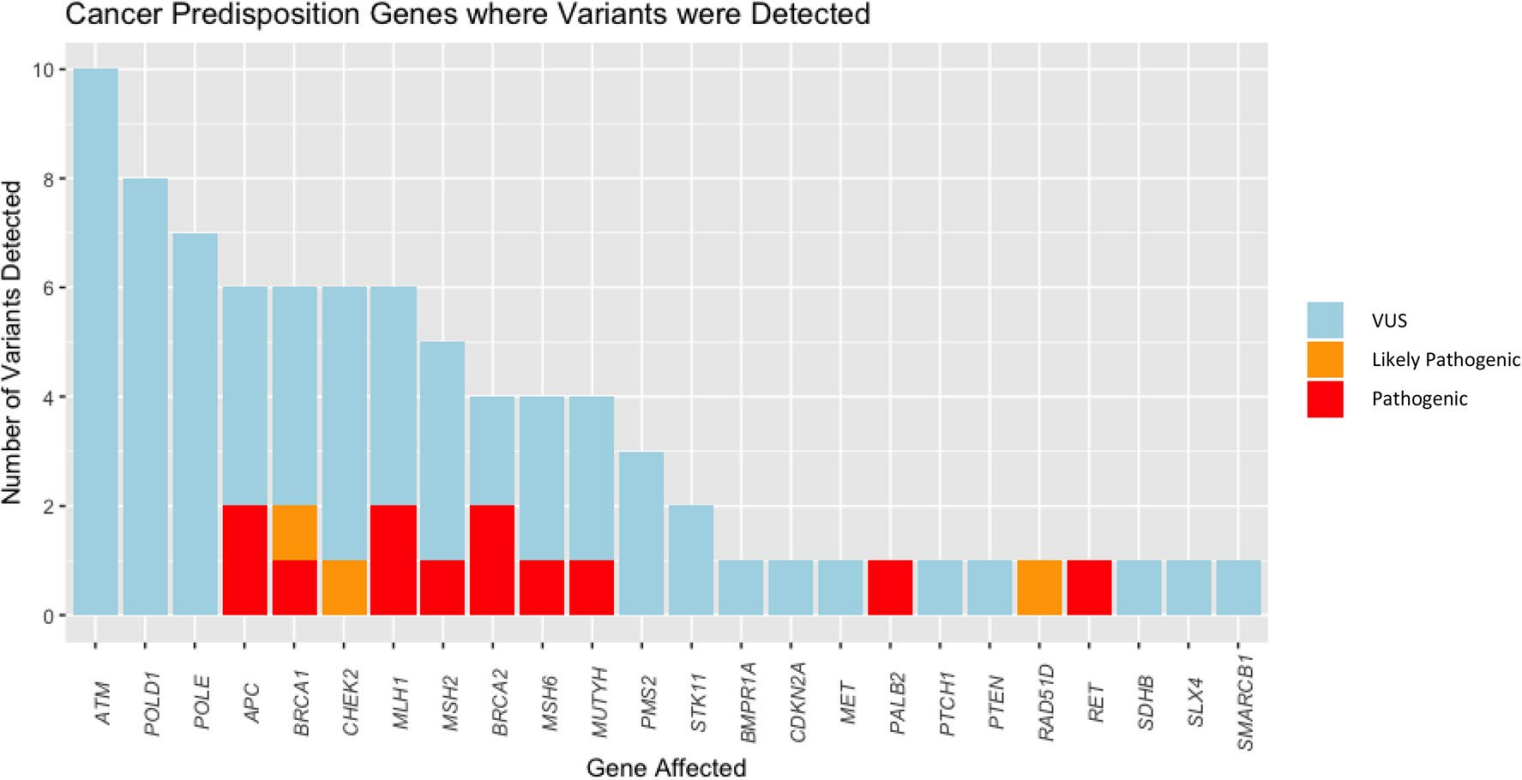
Cancer predisposition genes detected, sub-divided by the type of variant detected.

In 67 individuals, a total of 82 variants were detected in 24 known cancer predisposition genes (Fig. 3). 81.7% of variants were VUS (67 of 82), and 18.3% were P/LP (15 of 82). Focusing on the P/LP variants, these were detected in the following 11 cancer predisposition genes: *APC, BRCA1, BRCA2, CHEK2, MLH1, MSH2, MSH6, MUTYH, PALB2, RAD51D, RET*. All P/LP variants were actionable, warranting a change in care for both the patient and/or their family.

### Group comparisons

Using our demographic and variant data, we compared different groups to determine if there is a difference of statistical significance.

I. Individuals Affected by Cancer vs. Unaffected Individuals
  The VUS rate was similar (34% affected, 35% unaffected). In unaffected individuals undergoing testing, there was a higher P/LP rate (13%) compared to affected patients (8%), however this was not statistically significant with a Chi-square test p-value of 0.58.
II. Age of Cancer Onset in Affected Individuals
  The 98 patients undergoing diagnostic testing were divided into 2 groups: age of onset of cancer ≤ 50, and > 50, and the variants in each category were assessed. Associations between age and the following two variables were analysed: the number of P/LP variants observed, and the distribution of specific genes detected. Chi-square tests were both not statistically significant: *P* = 0.32 for the number of P/LP detected, and *P* = 0.55 for the distribution of the specific genes across age. In conclusion, there were no significant differences in gene panel results based on age at cancer onset in this study.
III. Fulfilment of Eligibility Criteria

**Figure 4:**
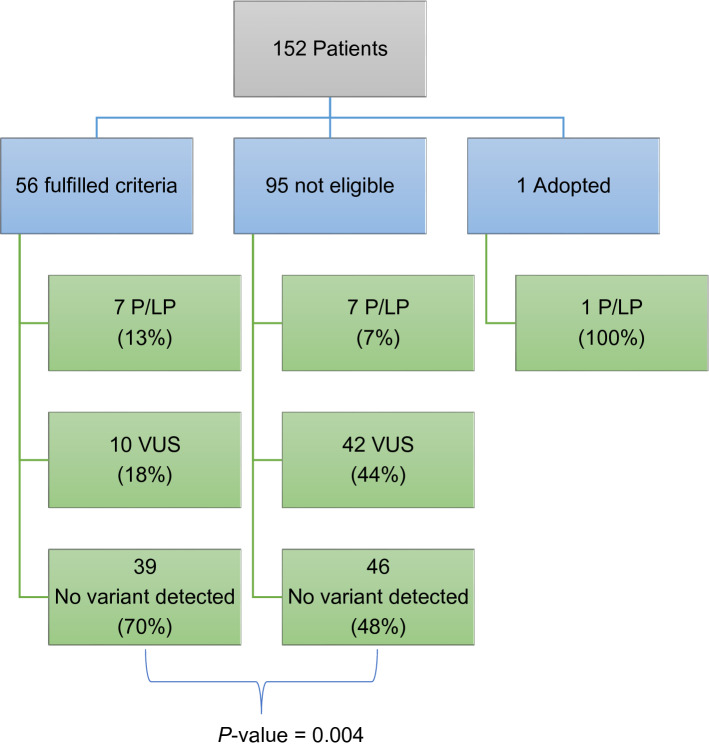
Overall results of gene panel testing, grouped by fulfilment of eligibility criteria. The individual who was adopted was excluded from this calculation as accurate estimation of family history was not possible.

All individuals in this study who underwent panel testing in the private sector were compared against eligibility criteria utilised by the NHS. 37% of patients (56 of 152) fulfilled NHS eligibility criteria. Summary statistics are displayed in Fig. 4. Comparing these two groups, there was a statistically significant difference in variant classification rates between those who were eligible for testing and those who were not, as expected.

### Focusing on the pathogenic/likely pathogenic variants

**Figure 5:**
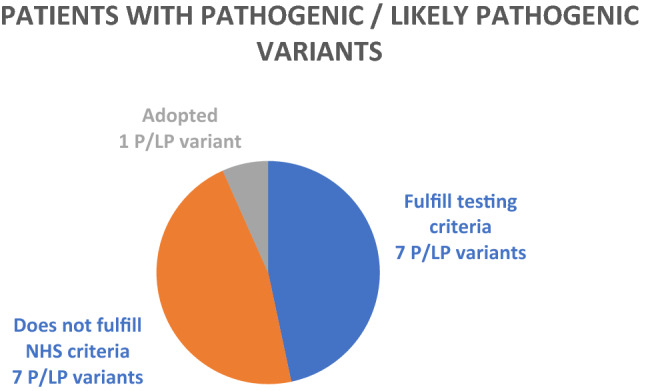
A detailed look at the patients with pathogenic / likely pathogenic variants: did they fulfil NHS eligibility criteria? In this study, the same number of P/LP variants were detected in patients who were eligible for genetic testing and those who were not.

Of the 15 P/LP variants detected by panel testing, 7 fulfilled criteria, 7 did not fulfil criteria, and 1 person was adopted. 46.7% of patients who received positive results in this study did not fulfil NHS testing criteria (Fig. 5).

*A closer look at the ****families**** of the 7 patients with P/LP variants who were not eligible for testing*: In 2 cases where an *APC* pathogenic variant was detected in patients who did not fulfil criteria, a different member of their family did. Conversely, the remaining 5 families, although there was a history of cancer, also did not fulfil testing criteria.

### Management of VUS outcomes

For the 52 individuals whose gene panel test yielded a VUS, all patients were informed and received pre and post-test counselling. The subject’s records were placed under annual review of VUS status. It is worth noting that some patients with VUS required additional cancer surveillance based on family history criteria, not the detection of a VUS. Over the 3-year period, only 1 VUS in *BRCA1* was re-classified to benign in a patient with triple negative breast cancer. The VUS rate in individuals from a non-European origin was 39%, versus 33% in Europeans.

### No P/LP variants were unexpected

Patients in whom P/LP variants were detected, the findings were consistent with either the personal or family history. There were 2 patients with breast cancer and heterozygous variants in *MLH1* and *MUTYH*. This, however, was consistent with their family histories of colorectal cancer.

### Clinical utility—Effect on the tested individual

**Figure 6:**
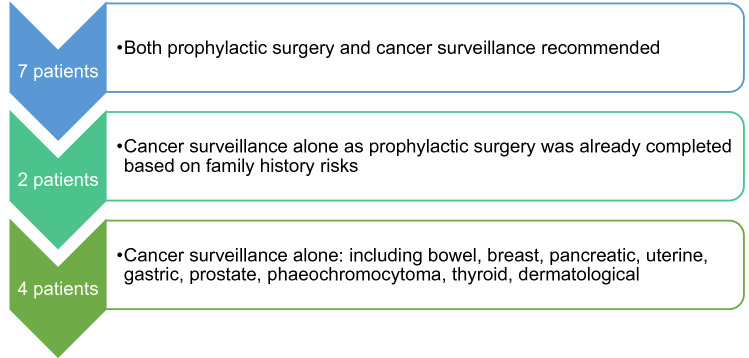
Actionability of gene panel results for 13 patients.

*13 of 15 P/LP results changed management for the patient* (Fig. 6). The first exception was a woman with breast cancer with a heterozygous pathogenic variant in MUTYH. Due to the autosomal recessive inheritance of the syndrome, neither prophylactic surgery nor cancer screening was recommended. However, the genetic testing did trigger predictive testing of her partner, due to implications for their child. The second exception was a patient in the palliative stages of advanced colorectal cancer.

### Clinical utility—Effect on the family

*15 of 15 P/LP results changed management for the families of the patients*. Members of all families were referred for predictive family testing and/or cancer surveillance. Clinical letters included documentation and counselling on implications for the family. One patient discussed the potential for pre-implantation genetic diagnosis with the physician.

## Discussion

### How does the rate of P/LP and VUS compare?

Of 152 patients, the variant detection rate was 10% for P/LP variants, and 34% for VUS. This is consistent with other researchers who have forgone testing criteria and achieved this variant threshold^2,3,30,35,40–44^. An example Tung et al.’s study on 488 women with breast cancer reports a P/LP rate of 10.7% and a VUS rate of 34%^41^. The P/LP frequency of 10% fulfils the threshold upon which many guidelines and family history-based criteria operate^5^.

All VUS detected in our study were reviewed yearly and patients were contacted by letter if any management was altered. Limitations of generalising this practice in the UK are that this degree of follow-up is not currently possible on the NHS. The additional resource burden associated with VUS may also entail familial segregation studies, tumour studies and functional testing. International academic institutions are working to define a standardised approach in clinical practice and policy^45^. Research groups are also endeavouring to assign degrees of risk of deleteriousness to VUS, which may aid clinical decision-making. Following the ACGS variant classification guidance published in 2020, the threshold for reporting VUS is now higher, and only ‘hot’ VUS should be reported after MDT discussion^46^. Current reporting policy offers promise in reducing the extent of the burden of VUS outcomes. Many VUS transpire to be benign on further follow-up.

### Eligibility criteria

Current tests offered in the NHS are restricted to patients who fulfil eligibility criteria. From our study, unrestricted testing of patients returns more actionable genetic variants than current eligibility criteria allow. Our study returned double the number of actionable genetic variants than NHS criteria. Overall, 7 additional P/LP variants were detected in the private clinic, in addition to the 7 that would have been detected on the NHS. Missing this proportion of P/LP variants (46.7%) is supported by prior research^47^.

Given that these eligibility criteria are evidence-based and designed to identify high-risk patients^48^, the higher P/LP detection rate and lower VUS rate in the group who fulfilled criteria was expected.

### Number needed to screen and risk reduction

**Figure 7:**
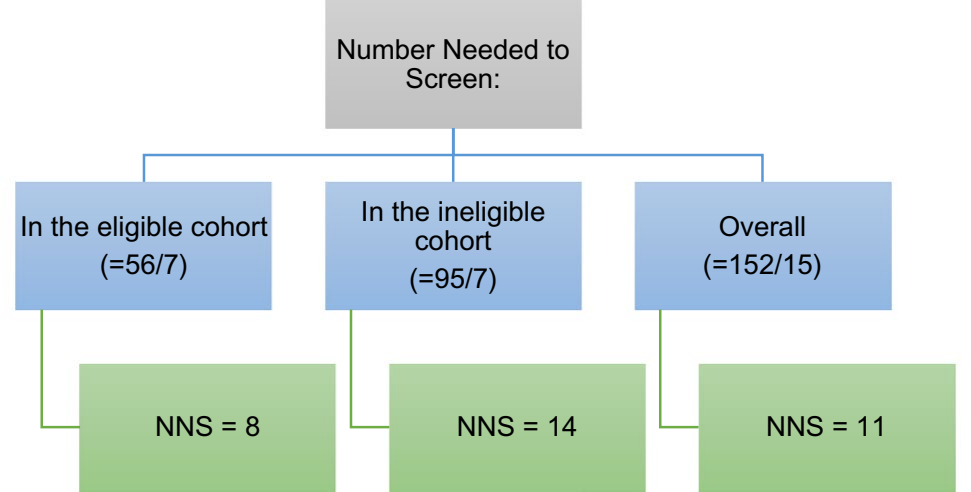
NNS for all patients, the eligible cohort, and the ineligible cohort.

Number needed to treat is a common statistical method used for assessing robustness of interventions, from drugs to screening programmes. A similar statistical method applicable to screening is the Number Needed to Screen (NNS)^49,50^. We applied this statistic to our data to identify how many patients need to undergo gene panel testing to find 1 P/LP variant. Results and calculations are shown in Fig. 7.

Although the same number of P/LP variants were found in both patients who fulfilled and did not fulfil NHS criteria, 6 additional patients were screened in the ineligible cohort. Overall, only 3 additional patients needed to be screened to find a P/LP variant.

We also tried to model to what extent additional testing is likely to improve survival. A large-scale meta-analysis conducted by Rebbeck et al. investigated the risk reduction of prophylactic salpingo-oophorectomy in carriers of P/LP variants in *BRCA1 and BRCA2*^51^. Prophylactic surgery conferred a reduction in breast cancer (hazard ratio = 0.49) and ovarian and fallopian tube cancers (hazard ratio = 0.21)^51^. In other words, there is a 79% risk reduction in ovarian and fallopian tube cancers in pathogenic *BRCA1/2* carriers with risk-reducing surgery. Utilising this data, as well as mortality figures of ovarian cancer in pathogenic *BRCA1/2* carriers, mortality is reduced 4.7-fold for *BRCA1*, and 46-fold for *BRCA2*. All calculations are demonstrated in Tables 3 and 4 for *BRCA1* and *BRCA2* respectively, and were based on the following assumptions: everyone will have had a prophylactic oophorectomy, patients have the same survival benefit as the documented literature, and all P/LP variants behave the same way.

**Table 3:** Calculating risk of death from ovarian cancer in P/LP ***BRCA1*** variant carriers.

	Before risk-reducing bilateral salpingo-oophorectomy	After risk-reducing bilateral salpingo-oophorectomy
Cumulative risk of ovarian cancer by age 70	39% ^52^	0.39 * 0.21 = 0.082 0.21 from Rebbeck et al. ^51^
Mortality hazard ratio in *BRCA1 carrier* regardless of tumour stage, grade, or histology	0.24 ^53^	0.24 ^53^
Overall risk of death	0.39 * 0.24 = 0.0936 = 9.4%	0.082 * 0.24 = 0.01968 = 2%

9.4% / 2% = 4.7-fold reduction in risk of death from ovarian cancer in P/LP BRCA1 carrier.

**Table 4:** Calculating risk of death from ovarian cancer in P/LP ***BRCA2*** variant carriers.

	Before risk-reducing bilateral salpingo-oophorectomy	After risk-reducing bilateral salpingo-oophorectomy
Cumulative risk of ovarian cancer by age 70	11% ^52^	0.11 * 0.21 = 0.0231 0.21 from Rebbeck et al. ^51^
Mortality hazard ratio in *BRCA2 carrier* regardless of tumour stage, grade, or histology	0.42 ^53^	0.42
Overall risk of death	0.11 * 0.42 = 0.0462 = 4.6%	0.0231 * 0.42 = 0.009702 = 0.1%

4.6% / 0.1% = 46-fold reduction in risk of death from ovarian cancer in P/LP BRCA1/2 carrier.

In this study, there were four P/LP *BRCA1/2* variants detected, one of whom was not eligible for testing and tested positive for a pathogenic *BRCA2* variant which would have otherwise been undetected. Her risk of death from ovarian cancer was reduced 46-fold according to the above calculation (Table 4).

### Unexpected findings

In two patients with breast cancer, heterozygous P/LP variants were detected in *MLH1* and *MUTYH*. Although a pattern of colorectal cancer was apparent in their family history, recent evidence does not define an association with breast cancer, except possibly in *MUTYH* carriers of two pathogenic alleles^54,55^. Both variants prompted cancer surveillance and/or family screening. An important note is that both of these genes are on the UK consensus of genes for medically-actionable conditions^1^. By and large, UK experts, drawing on their experience of the 100,000 Genomes Project, urge for clearer policy on the interpretation and reporting of secondary findings to patients^56^ and this is an avenue for future research.

### Clinical utility

The question posed here was as follows: will the variant alter clinical management compared with management based on family history alone? It is important to recognise that even without a detected variant, patients can be at an increased risk of cancer due to family history^27,28^. For example, if a 20-year-old woman has a first degree relative with breast cancer and an *ATM* pathogenic variant, and her own test is negative, her risk is still raised above the population^28^.

In our study, 87% (13 of 15) of results changed management for the patient. Even the two patients who had already received prophylactic surgery were additionally enrolled into cancer surveillance programmes. As aforementioned, the patients in which the results were not clinically actionable were a patient undergoing colorectal cancer treatment with a P/LP *MSH6* variant, and a heterozygous carrier of *MUTYH* (an autosomal recessive condition). However, 100% (all 15) of P/LP variants changed management for the families. This high rate of actionability is not discovered in all cancer gene panel studies^57^.

### Limitations

*Demographics*. As with any scientific study, this study too is not without limitations. These limitations are important to understand to enable critical review so that future studies may tackle what is left unanswered. The demographics are not representative of a general UK population: of 152 patients, 75.7% were female, and some ethnic minorities were over-represented. This is compounded by the small sample size. The skewed demographic may explain why common European P/LP variants, such as *CHEK2* 1100delC^30,58^, were not detected.

Additionally, we only included private patents from a single centre, and as such, local socioeconomic factors such as income and educational attainment could influence behaviour. Unlike on the NHS, all patients attending the centre were *actively seeking* testing. We also recognise that NHS eligibility criteria serve as guidance to healthcare professionals, and eligibility is also reviewed on a case-by-case basis^30^. Other differences in genetic testing between the NHS and private care include patient refusal, and not attending clinic appointments.

*Data Collection and Analysis*. We were limited by our reliance on family history and medical records which are not always complete. To evaluate psychosocial burdens, future studies including patient concern and satisfaction levels would be of value.

### Cancer gene panels: promise and pitfalls

#### The promise of cancer gene panel testing

Panels tests for germline testing have generated enthusiasm amongst clinical genetics services and increased our knowledge base of cancer genotype–phenotype associations^21,78^.

Gene panels offer the promise of time-efficiency by testing multiple genes simultaneously^59^, which is especially useful for genetically heterogeneous conditions^48,60^ and in patients who have previously tested negative in genetic tests^21^. Massively parallel sequencing is also cost-efficient utilising current advances in technology and requiring a smaller amount of DNA^1,6,21,61^.

Panel testing is paving a path towards the future of personalised medicine. Detecting clinically-relevant information through gene panels leads to early cancer detection and possible changes in management^45^, more so than conventional gene testing^30,62^. Risk stratification^32^ and clinical outcome prediction^31^ could be more accurate through the use of panel tests. Identification of variant status also has prognostic and therapeutic benefits. For example, carriers of P/LP *BRCA1/2* variants with breast cancer display sensitivity to platinum chemotherapy and PARP inhibitors^7^.

#### Pitfalls of cancer gene panels

The major challenge to gene panels is that our ability to interpret lags far behind our ability to sequence. The detection of VUS both in predictive and diagnostic testing renders the process more complex, both in terms of clinical management and counselling^45^. VUS have ramifications for clinical genetics services, with need for pre and post-test counselling, and our inability to explain ambiguous results of unknown deleteriousness to patients may cause more patient concern^45,63,64^. Studies on patient perspectives regarding gene panels have demonstrated their concerns about VUS rates, while recognising the potential positive impact on their health^65^, and the trust they place in healthcare professionals communicating genetic testing information^66^. The latter highlights the need for a more genome-educated medical community^64,66,67^, and dedicating resources to pre and post-test genetic counselling to help cope with the practical and psychological burdens associated with gene testing^21^.

Although standardisation of variant classification has proven difficult in the past^34,68,69^ The Cancer Variant Interpretation Group UK (CanVIG-UK) as well as the 2020 ACGS^46^ approach facilitate a standardised approach to classification and data sharing within NHS diagnostic labs. Internationally, the Clinical Genome (ClinGen) is striving to standardise approaches to variant classification, with gene-specific expert panels and a forum to resolve discrepant classifications.

There is also a need to standardise diagnostic reports on VUS to clarify to healthcare professionals the medical guidance associated with that variant^64^. Due to disparities in access to both research and genetic testing^12^, non-white ancestries have higher VUS rates^70^ as their reference panels are less well-defined, and ancestral branches differ in their genetic variation. Another area of ongoing research is the contribution of multiple variants to predisposition^29^.

VUS aside, detection of a P/LP variant is not always straightforward. If the variant is moderate risk, or is in a moderate-risk gene, parameters including risk estimates, phenotypic features, and actionability are not well-defined^29^. A discrepancy between genotype and phenotype may be detected, such as a moderate-risk gene in a high-risk family, or a highly pathogenic variant in a patient with unrelated symptoms. Such incidental or secondary findings are well described in the literature^6,30,31^. A well-known example is demonstrated by the Ohio Colorectal Cancer Prevention Initiative, where a high number of individuals with colorectal cancer had *BRCA1/2* variants^18^, despite evidence showing *BRCA1/2* does not confer a colorectal cancer risk.

Finally, there is concern that use of gene panels will raise ethical tensions, and large-scale implementation of gene panels needs to be both fair and appropriate. Factors to consider are financial sustainability, regulatory factors, and issues of equity^71,72^. At the rate at which gene tests are becoming available, it can be difficult to evaluate the genetic tests by the ACCE Framework, and their economic sustainability in universal healthcare systems such as the NHS^73^.

### Key points

Eligibility criteria attempt to strike a balance between identifying high-risk patients and minimising the level of uncertainty. We have demonstrated that cancer predisposition variants are not always associated with a significant family or personal history. Almost half of all patients with a P/LP result would not have fulfilled eligibility criteria for genetic testing and would therefore have been missed. Our results support a genetic testing policy that is less stringent, but testing for genes with a high degree of association with personal or family histories.

### Looking to the future: proceed, but with caution

There are multiple arguments to be made in favour of a broad approach to gene panel testing. Sequencing a larger number of affected and unaffected patients, then collating variants and associated features into a database will, in the long term, lead to enhanced classification of variants and understanding of their associated phenotypes across a multi-ethnic population^1,30,45^. Laboratories contribute coded germline findings to the Public Health England cancer registry, which is becoming an increasingly useful resource in practice, informing both prospective variant classification and assisting with review of existing classifications. The full potential of linking genetic data to the cancer registry has yet to be realised.

Limiting sequencing to patients with a strong personal or family history biases risk estimates, whereas a broad approach may reduce oversampling of severe cases and allow more reliable quantification of risks^32,79^. As our knowledge of individual variants and ability to call phenotypic consequences progresses, eligibility criteria may become more of a hindrance to both clinicians and patients^32^. Finally, we have yet to tap into the full scope of actionability of such variants. Development of targeted therapies^74,75^ and cancer screening algorithms based on genetic profiling^76^ are active areas of research.

There is disagreement on *how* to broaden our scope; how we go about simplifying access to genetic testing. This is an important area of future research. One approach is enhancing current policies on diagnostic testing of patients with cancer to enable more efficient and cost-effective cascade testing^26^. Delivering cancer predisposition gene testing through mainstreaming into routine oncological care would require remodelling of educational curricula and diagnostic clinical procedures, but can simplify access to genetic testing and benefit more patients^77^. The UK cancer genetics community is broadly in support of mainstreaming as specialist cancer genetics services do not have the capacity for the increasing demand^33^. Another approach is population-based screening to identify a cancer susceptibility gene, which may be a costly process, but it is argued to realise the true potential of screening^47^.

Our major bottleneck is accurate variant interpretation in the new era of big data. This bottleneck is the root of healthcare professionals’ hesitancy in embracing gene panels: ambiguous variant classification may trigger anxiety in patients, and at worst, lead to inappropriate tests and interventions^21^. The powerbrokers are bioinformaticians with an understanding of biology, or healthcare professionals and researchers equipped with the tools for understanding big data, who can transform raw primary sequences into variants with nuanced interpretations. This is another important area of future research.

To build a UK community of experts to deal with the influx in big genomic data, the Chief Medical Officer Professor Dame Sally Davies published her report ‘Generation Genome’, proposing some policies to lead the UK into the genomics era. These include centralised data analysis, harmonising genomics information across healthcare institutions, focusing on patient partnerships in patient-centric trials, and a focus on genomic education^67^. Some of these policies have sprouted with the 100,000 Genomes Project, helping us tap into the benefits of our single point-of-access system. Future local and regional UK-based research would provide a better idea of the current state, and where changes may be implemented.

There remain concerns about a less-restrictive policy which would need to be validated and reproduced prior to integration into clinical care. However, the only way to ensure the robustness of our data is to undertake more testing and validate this data. If validated, we have shown that for every 11 people tested, we find one P/LP variant which would alter management.

## Supplementary Information

Supplementary Information.

## Data Availability

The datasets generated during and analysed during the current study are not publicly available as although anonymised, they contain information that could in theory be identifiable. They are available from the corresponding author on reasonable request.

## References

[1] Taylor, A., Brady, A. F,, Frayling, I. M., Hanson, H., Tischkowitz, M., Turnbull, C., Side, L. & Group UKCG. Consensus for genes to be included on cancer panel tests offered by UK genetics services: Guidelines of the UK Cancer Genetics Group. *J. Med. Genet.* **16**, 16. 10.1136/jmedgenet-2017-105188 (2018). 10.1136/jmedgenet-2017-105188 PMC5992364 29661970

[2] Beitsch PD, Whitworth PW, Hughes K (2019). Underdiagnosis of hereditary breast cancer: Are genetic testing guidelines a tool or an obstacle?. J. Clin. Oncol..

[3] Samadder NJ, Riegert-Johnson D, Boardman L (2021). Comparison of universal genetic testing vs guideline-directed targeted testing for patients with hereditary cancer syndrome. JAMA Oncol..

[4] Whitworth J, Smith PS, Martin JE, West H, Luchetti A, Rodger F, Clark G, Carss K, Stephens J, Stirrups K, Penkett C, Mapeta R, Ashford S, Megy K, Shakeel H, Ahmed M, Adlard J, Barwell J, Brewer C, Casey RT, Armstrong R, Cole T, Evans DG, Fostira F, Greenhalgh L, Hanson H, Henderson A, Hoffman J, Izatt L, Kumar A, Kwong A, Lalloo F, Ong KR, Paterson J, Park SM, Chen-Shtoyerman R, Searle C, Side L, Skytte AB, Snape K, Woodward ER, Consortium NBRD, Tischkowitz MD, Maher ER (2018). Comprehensive cancer-predisposition gene testing in an adult multiple primary tumor series shows a broad range of deleterious variants and atypical tumor phenotypes. Am. J. Human Genet..

[5] National Collaborating Centre for Cancer (UK). Familial Breast Cancer: Classification and Care of People at Risk of Familial Breast Cancer and Management of Breast Cancer and Related Risks in People with a Family History of Breast Cancer. Cardiff (UK): National Collaborating Centre for Cancer (UK); 2013 Jun. (NICE Clinical Guidelines, No. 164.) Available from: https://www.ncbi.nlm.nih.gov/books/NBK247567/25340237

[6] Walsh T, Casadei S, Lee MK, Pennil CC, Nord AS, Thornton AM, Roeb W, Agnew KJ, Stray SM, Wickramanayake A, Norquist B, Pennington KP, Garcia RL, King MC, Swisher EM (2011). Mutations in 12 genes for inherited ovarian, fallopian tube, and peritoneal carcinoma identified by massively parallel sequencing. Proc. Natl. Acad. Sci. U.S.A..

[7] Rust K, Spiliopoulou P, Tang CY, Bell C, Stirling D, Phang T, Davidson R, Mackean M, Nussey F, Glasspool RM, Reed NS, Sadozye A, Porteous M, McGoldrick T, Ferguson M, Miedzybrodzka Z, McNeish IA, Gourley C (2018). Routine germline BRCA1 and BRCA2 testing in patients with ovarian carcinoma: analysis of the Scottish real-life experience. BJOG: Int. J. Obst. Gynaecol..

[8] Parkhurst E, Calonico E, Abboy S (2018). Utilization of genetic testing for RET mutations in patients with medullary thyroid carcinoma: A single-center experience. J. Genet. Couns..

[9] Romei C, Tacito A, Molinaro E, Agate L, Bottici V, Viola D, Matrone A, Biagini A, Casella F, Ciampi R, Materazzi G, Miccoli P, Torregrossa L, Ugolini C, Basolo F, Vitti P, Elisei R (2015). Twenty years of lesson learning: How does the RET genetic screening test impact the clinical management of medullary thyroid cancer?. Clin. Endocrinol..

[10] Alsop K, Fereday S, Meldrum C, deFazio A, Emmanuel C, George J, Dobrovic A, Birrer MJ, Webb PM, Stewart C, Friedlander M, Fox S (2012). Bowtell D and Mitchell G (2012) BRCA mutation frequency and patterns of treatment response in BRCA mutation-positive women with ovarian cancer: a report from the Australian Ovarian Cancer Study Group [Erratum appears in J Clin Oncol. 2012 Nov 20;30(33):4180]. J. Clin. Oncol..

[11] Wood ME, Kadlubek P, Pham TH, Wollins DS, Lu KH, Weitzel JN, Neuss MN, Hughes KS (2014). Quality of cancer family history and referral for genetic counseling and testing among oncology practices: A pilot test of quality measures as part of the American Society of Clinical Oncology Quality Oncology Practice Initiative. J. Clin. Oncol..

[12] Levy DE, Byfield SD, Comstock CB, Garber JE, Syngal S, Crown WH, Shields AE (2011). Underutilization of BRCA1/2 testing to guide breast cancer treatment: Black and Hispanic women particularly at risk. Genet. Med..

[13] Wright JD, Chen L, Tergas AI, Accordino M, Ananth CV, Neugut AI, Hershman DL (2016). Underuse of BRCA testing in patients with breast and ovarian cancer. Am. J. Obstet. Gynecol..

[14] Deanna Cross AR, Le A, Webster J, Potosky A, Feigelson H, Alexander G, Pawolski P, Williams A, Daida Y, Honda S, Onitilo A, McCarty C, Goddard K (2012). Lynch syndrome screening patterns in colorectal cancer patients in a large multi-institutional cohort. Current Med. Res..

[15] Lowery JT, Ahnen DJ, Schroy PC, Hampel H, Baxter N, Boland CR, Burt RW, Butterly L, Doerr M, Doroshenk M, Feero WG, Henrikson N, Ladabaum U, Lieberman D, McFarland EG, Peterson SK, Raymond M, Samadder NJ, Syngal S, Weber TK, Zauber AG, Smith R (2016). Understanding the contribution of family history to colorectal cancer risk and its clinical implications: A state-of-the-science review. Cancer.

[16] Febbraro T, Robison K, Wilbur JS, Laprise J, Bregar A, Lopes V, Legare R, Stuckey A (2015). Adherence patterns to National Comprehensive Cancer Network (NCCN) guidelines for referral to cancer genetic professionals. Gynecol. Oncol..

[17] Fiscella K, Winters P, Farah S, Sanders M, Mohile SG (2015). Do lung cancer eligibility criteria align with risk among Blacks and Hispanics?. PLoS ONE [Electronic Resource]..

[18] Pearlman, R., Frankel, W. L., Swanson, B., Zhao, W., Yilmaz, A., Miller, K., Bacher, J., Bigley, C., Nelsen, L., Goodfellow, P. J., Goldberg, R. M., Paskett, E., Shields, P. G., Freudenheim, J. L., Stanich, P. P., Lattimer, I., Arnold, M., Liyanarachchi, S., Kalady, M., Heald, B., Greenwood, C., Paquette, I., Prues, M., Draper, D. J., Lindeman, C., Kuebler, J. P., Reynolds, K., Brell, J. M., Shaper, A. A., Mahesh, S., Buie, N., Weeman, K., Shine, K., Haut, M., Edwards, J., Bastola, S., Wickham, K., Khanduja, K. S., Zacks, R., Pritchard, C. C., Shirts, B. H., Jacobson, A., Allen, B., de la Chapelle, A., Hampel, H. and Ohio Colorectal Cancer Prevention Initiative Study G. Prevalence and spectrum of germline cancer susceptibility gene mutations among patients with early-onset colorectal cancer. *JAMA Oncology.* **3**, 464–471. 10.1001/jamaoncol.2016.5194 (2016). 10.1001/jamaoncol.2016.5194 PMC5564179 27978560

[19] Fiederling J, Shams AZ, Haug U (2016). Validity of self-reported family history of cancer: A systematic literature review on selected cancers. Int. J. Cancer.

[20] Albright F, Stephenson RA, Agarwal N, Teerlink CC, Lowrance WT, Farnham JM, Albright LA (2015). Prostate cancer risk prediction based on complete prostate cancer family history. Prostate.

[21] Stanislaw C, Xue Y, Wilcox WR (2016). Genetic evaluation and testing for hereditary forms of cancer in the era of next-generation sequencing. Cancer Biol. Med..

[22] Augustinsson A, Ellberg C, Kristoffersson U, Borg A, Olsson H (2018). Accuracy of self-reported family history of cancer, mutation status and tumor characteristics in patients with early onset breast cancer. Acta Oncol..

[23] Lu, K. H., Wood, M. E., Daniels, M., Burke, C., Ford, J., Kauff, N. D., Kohlmann, W., Lindor, N. M., Mulvey, T. M., Robinson, L., Rubinstein, W. S., Stoffel, E. M., Snyder, C., Syngal, S., Merrill, J. K., Wollins, D. S., Hughes, K. S. and American Society of Clinical O. American Society of Clinical Oncology Expert Statement: Collection and use of a cancer family history for oncology providers. *J. Clin. Oncol.* **32**, 833–840. 10.1200/JCO.2013.50.9257 (2014). 10.1200/JCO.2013.50.9257 PMC3940540 24493721

[24] Mucci, L. A., Hjelmborg, J. B., Harris, J. R., Czene, K., Havelick, D. J., Scheike, T., Graff, R. E., Holst, K., Moller, S., Unger, R. H., McIntosh, C., Nuttall, E., Brandt, I., Penney, K. L., Hartman, M., Kraft, P., Parmigiani, G., Christensen, K., Koskenvuo, M., Holm, N. V., Heikkila, K., Pukkala, E., Skytthe, A., Adami, H. O., Kaprio, J. & Nordic Twin Study of Cancer C. Familial Risk and Heritability of Cancer Among Twins in Nordic Countries.[Erratum appears in JAMA. 2016 Feb 23;315(8):822; PMID: 26903347]. **1**, 68–76 (2016). 10.1001/jama.2015.17703 PMC5498110 26746459

[25] Anand P, Kunnumakkara AB, Sundaram C, Harikumar KB, Tharakan ST, Lai OS, Sung B, Aggarwal BB (2008). Cancer is a preventable disease that requires major lifestyle changes. Pharm. Res..

[26] Hampel H (2016). Genetic counseling and cascade genetic testing in Lynch syndrome. Fam. Cancer.

[27] Manolio TA, Collins FS, Cox NJ, Goldstein DB, Hindorff LA, Hunter DJ, McCarthy MI, Ramos EM, Cardon LR, Chakravarti A, Cho JH, Guttmacher AE, Kong A, Kruglyak L, Mardis E, Rotimi CN, Slatkin M, Valle D, Whittemore AS, Boehnke M, Clark AG, Eichler EE, Gibson G, Haines JL, Mackay TF, McCarroll SA, Visscher PM (2009). Finding the missing heritability of complex diseases. Nature.

[28] Lee AJ, Cunningham AP, Tischkowitz M, Simard J, Pharoah PD, Easton DF, Antoniou AC (2016). Incorporating truncating variants in PALB2, CHEK2, and ATM into the BOADICEA breast cancer risk model. Genet. Med..

[29] Stratton MR, Rahman N (2008). The emerging landscape of breast cancer susceptibility. Nat. Genet..

[30] Prapa M, Solomons J, Tischkowitz M (2017). The use of panel testing in familial breast and ovarian cancer. Clin. Med..

[31] Burke W (2014). Genetic tests: clinical validity and clinical utility. Current Protocols Human Genet..

[32] Easton DF, Pharoah PD, Antoniou AC, Tischkowitz M, Tavtigian SV, Nathanson KL, Devilee P, Meindl A, Couch FJ, Southey M, Goldgar DE, Evans DG, Chenevix-Trench G, Rahman N, Robson M, Domchek SM, Foulkes WD (2015). Gene-panel sequencing and the prediction of breast-cancer risk. N. Engl. J. Med..

[33] Slade I, Riddell D, Turnbull C, Hanson H, Rahman N, Programme MCG (2015). Development of cancer genetic services in the UK: A national consultation. Genome Med..

[34] Richards S, Aziz N, Bale S, Bick D, Das S, Gastier-Foster J, Grody WW, Hegde M, Lyon E, Spector E, Voelkerding K, Rehm HL, Committee ALQA (2015). Standards and guidelines for the interpretation of sequence variants: a joint consensus recommendation of the American College of Medical Genetics and Genomics and the Association for Molecular Pathology. Genet. Med..

[35] George A, Riddell D, Seal S, Talukdar S, Mahamdallie S, Ruark E, Cloke V, Slade I, Kemp Z, Gore M, Strydom A, Banerjee S, Hanson H, Rahman N (2016). Implementing rapid, robust, cost-effective, patient-centred, routine genetic testing in ovarian cancer patients. Sci. Rep..

[36] Manchanda R, Legood R, Burnell M, McGuire A, Raikou M, Loggenberg K, Wardle J, Sanderson S, Gessler S, Side L, Balogun N, Desai R, Kumar A, Dorkins H, Wallis Y, Chapman C, Taylor R, Jacobs C, Tomlinson I, Beller U, Menon U, Jacobs I (2015). Cost-effectiveness of population screening for BRCA mutations in Ashkenazi Jewish women compared with family history-based testing. J. Natl. Cancer Inst..

[37] Manchanda R, Patel S, Gordeev VS, Antoniou AC, Smith S, Lee A, Hopper JL, MacInnis RJ, Turnbull C, Ramus SJ, Gayther SA, Pharoah PDP, Menon U, Jacobs I, Legood R (2018). Cost-effectiveness of Population-Based BRCA1, BRCA2, RAD51C, RAD51D, BRIP1, PALB2 Mutation Testing in Unselected General Population Women. J. Natl. Cancer Inst..

[38] Kentwell M, Dow E, Antill Y, Wrede CD, McNally O, Higgs E, Hamilton A, Ananda S, Lindeman GJ, Scott CL (2017). Mainstreaming cancer genetics: A model integrating germline BRCA testing into routine ovarian cancer clinics. Gynecol. Oncol..

[39] National Genomic Test Directory. Testing Criteria for Rare and Inherited Disease. Available from: https://www.england.nhs.uk/publication/national-genomic-test-directories/.

[40] Yang S, Axilbund JE, O'Leary E, Michalski ST, Evans R, Lincoln SE, Esplin ED, Nussbaum RL (2018). Underdiagnosis of hereditary breast and ovarian cancer in medicare patients: Genetic testing criteria miss the mark. Ann. Surg. Oncol..

[41] Tung N, Lin NU, Kidd J, Allen BA, Singh N, Wenstrup RJ, Hartman AR, Winer EP, Garber JE (2016). Frequency of germline mutations in 25 cancer susceptibility genes in a sequential series of patients with breast cancer. J. Clin. Oncol..

[42] Gardner SA, Weymouth KS, Kelly WS, Bogdanova E, Chen W, Lupu D, Suhl J, Zeng Q, Geigenmuller U, Boles D, Okamoto PM, McDowell G, Hayden MA, Nagan N (2018). Evaluation of a 27-gene inherited cancer panel across 630 consecutive patients referred for testing in a clinical diagnostic laboratory. Hereditary Cancer Clin. Pract..

[43] Muller C, Nielsen SM, Hatchell KE, Yang S, Michalski ST, Hamlington B, Nussbaum RL, Esplin ED, Kupfer SS (2021). Underdiagnosis of hereditary colorectal cancers among medicare patients: genetic testing criteria for lynch syndrome miss the mark. JCO Precis Oncol..

[44] Uson PLS, Riegert-Johnson D, Boardman L, Kisiel J, Mountjoy L, Patel N, Lizaola-Mayo B, Borad MJ, Ahn D, Sonbol MB, Jones J, Leighton JA, Gurudu S, Singh H, Klint M, Kunze KL, Golafshar MA, Esplin ED, Nussbaum RL, Stewart AK, Bekaii-Saab TS, Jewel SN (2021). Germline cancer susceptibility gene testing in unselected patients with colorectal adenocarcinoma: A multicenter prospective study. Clin. Gastroenterol. Hepatol..

[45] Cheon JY, Mozersky J, Cook-Deegan R (2014). Variants of uncertain significance in BRCA: A harbinger of ethical and policy issues to come?. Genome Medicine..

[46] Ellard, S., Baple, E., Callaway, A., Berry, I., Forrester, N., Turnbull, C., Owens, M., Eccles, D. M., Abbs, S., Scott, R., Deans, Z. C., Lester, T., Campbell, J., Newman, W. G., Ramsden, S. & McMullan, D. J. ACGS Best Practice Guidelines for Variant Classification in Rare Disease 2020. https://www.acgs.uk.com/media/11631/uk-practice-guidelines-for-variant-classification-v4-01-2020.pdf.

[47] Foulkes WD, Knoppers BM, Turnbull C (2016). Population genetic testing for cancer susceptibility: founder mutations to genomes. Nat. Rev. Clin. Oncol..

[48] Kurian AW, Hare EE, Mills MA, Kingham KE, McPherson L, Whittemore AS, McGuire V, Ladabaum U, Kobayashi Y, Lincoln SE, Cargill M, Ford JM (2014). Clinical evaluation of a multiple-gene sequencing panel for hereditary cancer risk assessment. J. Clin. Oncol..

[49] Rembold CM (1998). Number needed to screen: development of a statistic for disease screening. BMJ.

[50] Auvinen A, Moss SM, Tammela TL, Taari K, Roobol MJ, Schroder FH, Bangma CH, Carlsson S, Aus G, Zappa M, Puliti D, Denis LJ, Nelen V, Kwiatkowski M, Randazzo M, Paez A, Lujan M, Hugosson J (2016). Absolute effect of prostate cancer screening: Balance of benefits and harms by center within the european randomized study of prostate cancer screening [Erratum appears in Clin Cancer Res. 2016 Jul 15;22(14):3702; PMID: 27422205]. Clin. Cancer Res..

[51] Rebbeck TR, Kauff ND, Domchek SM (2009). Meta-analysis of risk reduction estimates associated with risk-reducing salpingo-oophorectomy in BRCA1 or BRCA2 mutation carriers. J. Natl Cancer Inst..

[52] Antoniou A, Pharoah PD, Narod S, Risch HA, Eyfjord JE, Hopper JL, Loman N, Olsson H, Johannsson O, Borg A, Pasini B, Radice P, Manoukian S, Eccles DM, Tang N, Olah E, Anton-Culver H, Warner E, Lubinski J, Gronwald J, Gorski B, Tulinius H, Thorlacius S, Eerola H, Nevanlinna H, Syrjakoski K, Kallioniemi OP, Thompson D, Evans C, Peto J, Lalloo F, Evans DG, Easton DF (2003). Average risks of breast and ovarian cancer associated with BRCA1 or BRCA2 mutations detected in case Series unselected for family history: a combined analysis of 22 studies [Erratum appears in Am J Hum Genet. 2003 Sep;73(3):709]. Am. J. Human Genet..

[53] Zhong Q, Peng HL, Zhao X, Zhang L, Hwang WT (2015). Effects of BRCA1- and BRCA2-related mutations on ovarian and breast cancer survival: A meta-analysis. Clin. Cancer Res..

[54] Rennert G, Lejbkowicz F, Cohen I, Pinchev M, Rennert HS, Barnett-Griness O (2012). MutYH mutation carriers have increased breast cancer risk. Cancer.

[55] Out AA, Wasielewski M, Huijts PE, van Minderhout IJ, Houwing-Duistermaat JJ, Tops CM, Nielsen M, Seynaeve C, Wijnen JT, Breuning MH, van Asperen CJ, Schutte M, Hes FJ, Devilee P (2012). MUTYH gene variants and breast cancer in a Dutch case-control study. Breast Cancer Res. Treat..

[56] Ormondroyd E, Mackley MP, Blair E, Craft J, Knight JC, Taylor JC, Taylor J, Watkins H (2018). "Not pathogenic until proven otherwise": Perspectives of UK clinical genomics professionals toward secondary findings in context of a Genomic Medicine Multidisciplinary Team and the 100,000 Genomes Project. Genet. Med..

[57] Johns AL, McKay SH, Humphris JL, Pinese M, Chantrill LA, Mead RS, Tucker K, Andrews L, Goodwin A, Leonard C, High HA, Nones K, Patch AM, Merrett ND, Pavlakis N, Kassahn KS, Samra JS, Miller DK, Chang DK, Pajic M, Australian Pancreatic Cancer Genome I, Pearson JV, Grimmond SM, Waddell N, Zeps N, Gill AJ, Biankin AV (2017). Lost in translation: returning germline genetic results in genome-scale cancer research. Genome Med..

[58] Consortium CBCC-C. CHEK2 1100delC and susceptibility to breast cancer: A collaborative analysis involving 10,860 breast cancer cases and 9,065 controls from 10 studies. *Am. J. Human Genet.*** 74**, 1175–1182 (2004). 10.1086/421251 PMC1182081 15122511

[59] Buys SS, Sandbach JF, Gammon A, Patel G, Kidd J, Brown KL, Sharma L, Saam J, Lancaster J, Daly MB (2017). A study of over 35,000 women with breast cancer tested with a 25-gene panel of hereditary cancer genes. Cancer.

[60] LaDuca H, Stuenkel AJ, Dolinsky JS, Keiles S, Tandy S, Pesaran T, Chen E, Gau CL, Palmaer E, Shoaepour K, Shah D, Speare V, Gandomi S, Chao E (2014). Utilization of multigene panels in hereditary cancer predisposition testing: Analysis of more than 2,000 patients. Genet. Med..

[61] Payne K, Gavan SP, Wright SJ, Thompson AJ (2018). Cost-effectiveness analyses of genetic and genomic diagnostic tests. Nat. Rev. Genet..

[62] Desmond A, Kurian AW, Gabree M, Mills MA, Anderson MJ, Kobayashi Y, Horick N, Yang S, Shannon KM, Tung N, Ford JM, Lincoln SE, Ellisen LW (2015). Clinical actionability of multigene panel testing for hereditary breast and ovarian cancer risk assessment. JAMA Oncol..

[63] Domchek SM, Bradbury A, Garber JE, Offit K, Robson ME (2013). Multiplex genetic testing for cancer susceptibility: Out on the high wire without a net?. J. Clin. Oncol..

[64] Eccles BK, Copson E, Maishman T, Abraham JE, Eccles DM (2015). Understanding of BRCA VUS genetic results by breast cancer specialists. BMC Cancer.

[65] Frost CJ, Andrulis IL, Buys SS, Hopper JL, John EM, Terry MB, Bradbury A, Chung WK, Colbath K, Quintana N, Gamarra E, Egleston B, Galpern N, Bealin L, Glendon G, Miller LP, Daly MB (2018). Assessing patient readiness for personalized genomic medicine. J. Community Genet..

[66] Roberts MC, Taber JM, Klein WM (2018). Engagement with genetic information and uptake of genetic testing: The role of trust and personal cancer history. J. Cancer Educ..

[67] Davies S. *Chief Medical Officer annual report 2016: Generation Genome*. Department of Health and Social Care.

[68] Amendola LM, Jarvik GP, Leo MC, McLaughlin HM, Akkari Y, Amaral MD, Berg JS, Biswas S, Bowling KM, Conlin LK, Cooper GM, Dorschner MO, Dulik MC, Ghazani AA, Ghosh R, Green RC, Hart R, Horton C, Johnston JJ, Lebo MS, Milosavljevic A, Ou J, Pak CM, Patel RY, Punj S, Richards CS, Salama J, Strande NT, Yang Y, Plon SE, Biesecker LG, Rehm HL (2016). Performance of ACMG-AMP variant-interpretation guidelines among nine laboratories in the clinical sequencing exploratory research consortium [Erratum for Am J Hum Genet. 2016 Jun 2;98 (6):1067–1076; PMID: 27181684]. Am. J. Human Genet..

[69] Balmana J, Digiovanni L, Gaddam P, Walsh MF, Joseph V, Stadler ZK, Nathanson KL, Garber JE, Couch FJ, Offit K, Robson ME, Domchek SM (2016). Conflicting Interpretation of genetic variants and cancer risk by commercial laboratories as assessed by the prospective registry of multiplex testing. J. Clin. Oncol..

[70] Eggington JMBL, Roa B, Pruss D, Bowles K, Rosenthal E, Esterling L, Wenstrup R (2012). Current Variant of Uncertain Significance Rates in BRCA1/2 and Lynch Syndrome Testing (MLH1, MSH2, MSH6, PMS2, EPCAM).

[71] Botkin JR, Belmont JW, Berg JS, Berkman BE, Bombard Y, Holm IA, Levy HP, Ormond KE, Saal HM, Spinner NB, Wilfond BS, McInerney JD (2015). Points to consider: Ethical, legal, and psychosocial implications of genetic testing in children and adolescents. Am. J. Hum. Genet..

[72] Minari J, Brothers KB, Morrison M (2018). Tensions in ethics and policy created by National Precision Medicine Programs. Hum. Genomics.

[73] Pitini E, De Vito C, Marzuillo C, D'Andrea E, Rosso A, Federici A, Di Maria E, Villari P (2018). How is genetic testing evaluated? A systematic review of the literature. Eur. J. Hum. Genet..

[74] Diaz LA, Le DT (2015). PD-1 blockade in tumors with mismatch-repair deficiency. N. Engl. J. Med..

[75] Le DT, Durham JN, Smith KN, Wang H, Bartlett BR, Aulakh LK, Lu S, Kemberling H, Wilt C, Luber BS, Wong F, Azad NS, Rucki AA, Laheru D, Donehower R, Zaheer A, Fisher GA, Crocenzi TS, Lee JJ, Greten TF, Duffy AG, Ciombor KK, Eyring AD, Lam BH, Joe A, Kang SP, Holdhoff M, Danilova L, Cope L, Meyer C, Zhou S, Goldberg RM, Armstrong DK, Bever KM, Fader AN, Taube J, Housseau F, Spetzler D, Xiao N, Pardoll DM, Papadopoulos N, Kinzler KW, Eshleman JR, Vogelstein B, Anders RA, Diaz LA (2017). Mismatch repair deficiency predicts response of solid tumors to PD-1 blockade. Science.

[76] Dias A, Kote-Jarai Z, Mikropoulos C, Eeles R (2017). Prostate cancer germline variations and implications for screening and treatment. Cold Spring Harb. Perspect. Med..

[77] Rahman N (2014). Mainstreaming genetic testing of cancer predisposition genes. Clin. Med..

[78] Breast Cancer Association Consortium (2021). Breast cancer risk genes - association analysis in more than 113,000 women. N. Engl. J. Med..

[79] Hu C, Hart S, Gnanaolivu R, Huang H, Lee K, Na J, Gao C, Lilyquist J, Yadav S, Boddicker N, Samara R, Klebba J (2021). A population-based study of genes previously implicated in breast cancer. N. Engl. J. Med..

