# Supplementary material for: Half of germline pathogenic and likely pathogenic variants found on panel tests do not fulfil NHS testing criteria
Source: Sci Rep. 2022 Feb 21;12:2507. doi: 10.1038/s41598-022-06376-4 (PMC8861039; doi:10.1038/s41598-022-06376-4)
Supplement: Supplementary file 1 — Supplementary Information. [file 41598_2022_6376_MOESM1_ESM.docx]

Supplementary Table 1: Genes tested for each patient

| *Patient Number* | *Report Interpretation* |
| --- | --- |
| 1 | *APC, BMPR1A, BRCA1, BRCA2, HOXB13, MLH1, MSH2, MSH6, MUTYH, PMS2, POLD1, POLE, PTEN, SMAD4, STK11, TP53* |
| 2 | *AIP, APC, ATM, BARD1, BMPR1A, BRCA1, BRCA2, BRIP1, CASR, CDC73, CDH1, CDKN1B, CDKN2A, CHEK2, ERCC4, FANCA, FANCB, FANCC, FANCD2, FANCE, FANCF, FANCG, FANCI, FANCL, FANCM, GALNT12, GREM1, KIT, MEN1, MLH1, MSH2, MSH6, MUTYH, NF1, PALB2, PIK3CA, PMS2, POLD1, POLE, PRKAR1A, PTCH1, PTEN, RAD51C, RAD51D, RB1, RET, SLX4, SMAD4, SMARCB1, SPRED1, STK11, TP53* |
| 3 | *APC, BMPR1A, BRCA1, BRCA2, BRIP1, CDH1, CDK4, CDKN2A, CHEK2, CTRC, MLH1, MSH2, MSH6, MUTYH, PALB2, PMS2, POLD1, POLE, PTEN, RAD51C, RAD51D, RET, SMAD4, SPINK1, STK11, TP53* |
| 4 | *ATM, BRCA1, BRCA2, CHEK2, MLH1, MSH2, MSH6, PALB2, PMS2, PTEN, STK11, TP53* |
| 5 | *ATM, BRCA1, BRCA2, CDH1, CHEK2, MLH1, MSH2, MSH6, NF1, PALB2, PMS2, PTEN, STK11, TP53* |
| 6 | *APC, ATM, BMPR1A, BRCA1, BRCA2, CHEK2, MLH1, MSH2, MSH6, MUTYH, NF1, PALB2, PMS2, POLD1, POLE, PTEN, SMAD4, STK11, TP53* |
| 7 | *ATM, BRCA1, BRCA2, BRIP1, CHEK2, MLH1, MSH2, MSH6, PALB2, PMS2, PTEN, RAD51C, RAD51D, STK11, TP53* |
| 8 | *ATM, BRCA1, BRCA2, BRIP1, CDH1, CHEK2, ERCC4, FANCA, FANCB, FANCC, FANCD2, FANCE, FANCF, FANCG, FANCI, FANCL, FANCM, MLH1, MSH2, MSH6, PALB2, PMS2, PTEN, RAD51C, RAD51D, STK11, TP53* |
| 9 | *ATM, BARD1, BRCA1, BRCA2, BRIP1, CHEK2, MLH1, MSH2, MSH6, NF1, PALB2, PMS2, PTEN, RAD51C, RAD51D, STK11 & TP53* |
| 10 | *APC, ATM, BMPR1A, BRCA1, BRCA2, CDH1, MLH1, MSH2, MSH6, MUTYH, NF1, PALB2, PMS2, POLD1, POLE, PTEN, SMAD4, STK11, TP53* |
| 11 | *ATM, BRCA1, BRCA2, BRIP1, CHEK2, MLH1, MSH2, MSH6, PALB2, PMS2, PTEN, RAD51C, RAD51D, STK11, TP53* |
| 12 | *APC, BMPR1A, BRCA1, BRCA2, CHEK2, HOXB13, MLH1, MSH2, MSH6, MUTYH, PALB2, PMS2, POLD1, POLE, PTEN, SMAD4, STK11, TP53* |
| 13 | *ATM, BRCA1, BRCA2, BRIP1, CDH1, CHEK2, MLH1, MSH2, MSH6, NF1, PALB2, PMS2, PTEN, RAD51C, RAD51D, STK11, TP53* |
| 14 | *APC, ATM, BMPR1A, BRCA1, BRCA2, MLH1, MSH2, MSH6, MUTYH, PALB2, PMS2, POLD1, POLE, PTEN, SMAD4, STK11, TP53* |
| 15 | *APC, ATM, BMPR1A, BRCA1, BRCA2, MLH1, MSH2, MSH6, MUTYH, PALB2, PMS2, POLD1, POLE, PTEN, SMAD4, STK11, TP53* |
| 16 | *ATM, BRCA1, BRCA2, CDH1, CDK4, CDKN2A, CHEK2, MLH1, MSH2, MSH6, NF1, PALB2, PMS2, PTEN, RAD51C, RAD51D, SMAD4, STK11, TP53* |
| 17 | *ATM, BRCA1, BRCA2, BRIP1, CHEK2, HOXB13, MLH1, MSH2, MSH6, PALB2, PMS2, PTEN, RAD51C, RAD51D, STK11, TP53* |
| 18 | *ATM, BRCA1, BRCA2, CDH1, CHEK2, MLH1, MSH2, MSH6, NF1, PALB2, PMS2, PTEN, STK11, TP53* |
| 19 | *BRCA1, BRCA2, CDK4, CDKN2A (p16), CDKN2A (p14), MLH1, MSH2, MSH6, PMS2, PTEN, STK11, TP53* |
| 20 | *APC, BMPR1A, BRCA1, BRCA2, CDK4, CDKN2A, MLH1, MSH2, MSH6, MUTYH, PMS2, POLD1, POLE, PTCH1, PTEN, RET, SMAD4, STK11, TP53* |
| 21 | *BRCA1, BRCA2, BRIP1, NF1, PALB2, PTEN, RAD51C, RAD51D, STK11, TP53* |
| 22 | *APC, BMPR1A, BRCA1, BRCA2, CDH1, CDK4, CDKN2A, CHEK2, MLH1, MSH2, MSH6, MUTYH, PMS2, POLD1, POLE, PTEN, SMAD4, STK11, TP53* |
| 23 | *BRCA1+B26, BRCA2, CDH1, CHEK2, PTEN, STK11, TP53* |
| 24 | *APC, ATM, BMPR1A, CDH1, CHEK2, FH, MLH1, MSH2, MSH6, MUTYH, PALB2, POLD1, POLE, PTEN, RAD51C, SLX4, SMAD4, STK11, TP53* |
| 25 | *ATM, BRCA1, BRCA2, BRIP1, CDH1, CHEK2, MLH1, MSH2, MSH6, NF1, PALB2, PMS2, PTEN, RAD51C, RAD51D, STK11, TP53* |
| 26 | *BRCA1, BRCA2, HOXB13, MLH1, MSH2, MSH6* |
| 27 | *This patient has been screened for mutations in the entire set of 80 cancer predisposing genes on the Leeds Panel by sequence and dosage analysis.* |
| 28 | *BRCA1, BRCA2, CDH1, DICER1, PTEN, STK11, TP53* |
| 29 | *APC, BMPR1A, BRCA1, BRCA2, MLH1, MSH2, MSH6, MUTYH, PMS2, PTEN, SMAD4, STK11, TP53* |
| 30 | *APC, BMPR1A, MLH1, MSH2, MSH6, MUTYH, PMS2, POLD1, POLE, SMAD4, STK11* |
| 31 | *BRCA1, BRCA2, MLH1, MSH2, MSH6, PMS2, TP53* |
| 32 | *APC, ATM, BRCA1, BRCA2, CDH1, MLH1, MSH2, MSH6, NF1, PMS2, PTCH1, PTEN, STK11, TP53* |
| 33 | *APC, BMPR1A, BRCA1, BRCA2, CDH1, CHEK2, MLH1, MSH2, MSH6, MUTYH, PMS2, POLD1, POLE, PTEN, SMAD4, STK11, TP53* |
| 34 | *APC, BMPR1A, CDH1, MLH1, MSH2, MSH6, MUTYH, PMS2, POLD1, POLE, SMAD4, STK11* |
| 35 | *BARD1, BRCA1, BRCA2, BRIP1, MLH1, MSH2, MSH6, PALB2, PMS2, PTEN, RAD51C, RAD51D, STK11, TP53* |
| 36 | *APC, BMPR1A, BRCA1, BRCA2, CHEK2, MLH1, MSH2, MSH6, MUTYH, PMS2, PTEN, SMAD4, STK11, TP53* |
| 37 | *ATM, BRCA1, BRCA2, CDH1, CHEK2, HOXB13, MLH1, MSH2, MSH6, MUTYH, NF1, PALB2, PMS2, PTEN, RAD51C, RAD51D, RET, STK11, TP53* |
| 38 | *BRCA1, BRCA2, CDH1, CHEK2, PALB2, PTEN, STK11* |
| 39 | *BRCA1, BRCA2, RET* |
| 40 | *APC, ATM, BMPR1A, CDK4, CDKN2A, CHEK2, CTRC, HOXB13, MLH1, MSH2, MSH6, MUTYH, NF1, PALB2, PMS2, POLD1, POLE, PTEN, SMAD4, SPINK1, STK11, TP53* |
| 41 | *APC, ATM, BMPR1A, BRCA1, BRCA2, BRIP1, CHEK2, MUTYH, NF1, PALB2, PMS2, POLD1, POLE, PTEN, RAD51C, RAD51D, SMAD4, STK11, TP53* |
| 42 | *BRCA1, BRCA2, CDH1, CDK4, CDKN2A, PTEN, STK11, TP53* |
| 43 | *APC, BMPR1A, BRCA1, BRCA2, CDH1, CDK4, CDKN2A, CHEK2, MLH1, MSH2, MSH6, MUTYH, PMS2, POLD1, POLE, PTEN, SMAD4, STK11, TP53* |
| 44 | *APC, ATM, BMPR1A, BRCA1, BRCA2, CHEK2, HOXB13, MLH1, MSH2, MSH6, MUTYH, PALB2, PMS2, POLD1, POLE, PTCH1, PTEN, SMAD4, STK11, TP53* |
| 45 | *APC, BMPR1A, BRCA1, BRCA2, CDH1, CHEK2, MLH1, MSH2, MSH6, MUTYH, PMS2, PTEN, SMAD4, STK11, TP53* |
| 46 | *ATM, BRCA1, BRCA2, CHEK2, MLH1, MSH2, MSH6, NF1, PALB2, PMS2, PTEN, STK11, TP53* |
| 47 | *ATM, BRCA1, BRCA2, BRIP1, CDK4, CDKN2A, CHEK2, CTRC, MLH1, MSH2, MSH6, NF1, PALB2, PMS2, PTEN, RAD51C, RAD51D, SPINK1, STK11, TP53* |
| 48 | *BRCA1, BRCA2, PTEN, STK11, TP53* |
| 49 | *APC, BMPR1A, MLH1, MSH2, MSH6, MUTYH, PMS2, POLD1, POLE, SMAD4, STK11* |
| 50 | *MAX, NF1, PRKAR1A, RET, SDHA, SDHAF2, SDHB, SDHC, SDHD, TMEM127, VHL* |
| 51 | *APC, BMPR1A, BRCA1, BRCA2, CDK4, CDKN2A, CHEK2, MLH1, MSH2, MSH6, MUTYH, PMS2, POLD1, POLE, SMAD4, STK11, TP53* |
| 52 | *BRCA1, BRCA2, FH, FLCN, MET, MLH1, MSH2, MSH6, PMS2, PTEN, SDHB, STK11, TP53, VHL* |
| 53 | *BRCA1 & BRCA2 also APC, BMPR1A, CDK4, CDKN2A, MLH1, MSH2, MSH6, MUTYH, PMS2, PTEN, SMAD4, STK11, TP53* |
| 54 | *APC, ATM, BMPR1A, BRCA1, BRCA2, CHEK2, FH, FLCN, MET, MLH1, MSH2, MSH6, MUTYH, NF1, PALB2, PMS2, POLD1, POLE, PTEN, SDHB, SMAD4, STK11, TP53, VHL* |
| 55 | *BRIP1, RAD51C, RAD51D & BMPR1A, BRCA1, BRCA2, CDH1, MLH1, MSH2, MSH6, PMS2, PTEN, STK11, TP53* |
| 56 | *ATM, BRCA1, BRCA2, BRIP1, CDH1, CHEK2, MLH1, MSH2, MSH6, MUTYH, PALB2, PMS2, PTEN, RAD51C, RAD51D, STK11, TP53* |
| 57 | *APC, BMPR1A, MLH1, MSH2, MSH6, MUTYH, PMS2, POLD1, POLE, SMAD4, STK11* |
| 58 | *ATM, BRCA1, BRCA2, BRIP1, CHEK2, HOXB13, MLH1, MSH2, MSH6, MUTYH, PALB2, PMS2, PTEN, STK11, TP53* |
| 59 | *APC, BMPR1A, BRCA1, BRCA2, CDH1, CDK4, CDKN2A, CHEK2, MLH1, MSH2, MSH6, MUTYH, PMS2, POLD1, POLE, PTEN, SMAD4, STK11, TP53* |
| 60 | *ATM, BRCA1, BRCA2, CHEK2, MLH1, MSH2, MSH6, PALB2, PMS2, PTEN, RAD51C, RAD51D, STK11, TP53* |
| 61 | *BMPR1A, BRCA1, BRCA2, CDH1, MLH1, MSH2, MSH6, PALB2, PMS2, PTEN, SMAD4, STK11, TP53* |
| 62 | *CDH1, CDK4, CDKN2A, FH, FLCN, MET, NF1, NF2, PTEN, SDHB, SMARCB1, SMARCE1, SPRED1, STK11, TP53, VHL* |
| 63 | *APC, BMPR1A, BRCA1, BRCA2, CDH1, HOXB13, MLH1, MSH2, MSH6, MUTYH, PMS2, POLD1, POLE, PTEN, SMAD4, STK11, TP53* |
| 64 | *APC, BMPR1A, BRCA1, BRCA2, CDH1, CDK4, CDKN2A, CHEK2, MLH1, MSH2, MSH6, MUTYH, PMS2, POLD1, POLE, PTEN, SMAD4, STK11, TP53* |
| 65 | *APC, BMPR1A, BRCA1, BRCA2, CDH1, FH, MLH1, MSH2, MSH6, MUTYH, PMS2, PTEN, SMAD4, STK11, TP53* |
| 66 | *APC, BMPR1A, MLH1, MSH2, MSH6, MUTYH, PMS2, POLD1, POLE, SMAD4, STK11* |
| 67 | *ATM, BRCA1, BRCA2, BRIP1, CHEK2, FH, FLCN, HOXB13, MET, MLH1, MSH2, MSH6, PMS2, PTEN, SDHB, STK11, TP53, VHL* |
| 68 | *APC, BMPR1A, BRCA1, BRCA2, MLH1, MSH2, MSH6, MUTYH, PMS2, PTCH1, PTEN, SMAD4, STK11, TP53* |
| 69 | *ATM, BAP1, BRCA1, BRCA2, CDK4, CDKN2A, MLH1, MSH2, MSH6, NF1, PMS2, RB1, SMARCB1, TP53* |
| 70 | *ATM, BRCA1, BRCA2, BRIP1, CHEK2, MLH1, MSH2, MSH6, NF1, PALB2, PMS2, PTEN, RAD51C, RAD51D, STK11, TP53* |
| 71 | *APC, BMPR1A, BRCA1, BRCA2, FH, FLCN, HOXB13, MET, MLH1, MSH2, MSH6, MUTYH, PMS2, POLD1, POLE, PTEN, SDHB, SMAD4, STK11, TP53, VHL* |
| 72 | *BRCA1, BRCA2, CDH1, CDK4, CDKN2A, CTRC, HOXB13, MLH1, MSH2, MSH6, PALB2, PMS2, PTEN, SPINK1, STK11, TP53* |
| 73 | *APC, BMPR1A, BRCA1, BRCA2, MLH1, MSH2, MSH6, MUTYH, PMS2, POLD1, POLE, PTCH1, PTEN, SMAD4, STK11, TP53* |
| 74 | *BRCA1, BRCA2, CDK4, CDKN2A, FH, MLH1, MSH2, MSH6, PALB2, PMS2, PTEN, STK11, TP53* |
| 75 | *ATM, BRCA1, BRCA2, CDH1, CHEK2, MLH1, MSH2, MSH6, NF1, PALB2, PMS2, PTEN, STK11, TP53* |
| 76 | *APC, BMPR1A, BRCA1, BRCA2, CDH1, MLH1, MSH2, MSH6, MUTYH, PMS2, POLD1, POLE, PTEN, SMAD4, STK11, TP53* |
| 77 | *BRCA1, BRCA2, CDH1, PTEN, RB1, STK11, TP53* |
| 78 | *APC, ATM, BMPR1A, BRCA1, BRCA2, BRIP1, CDH1, CDK4, CDKN2A, CHEK2, ERCC4, FANCA, FANCB, FANCC, FANCD2, FANCE, FANCF, FANCG, FANCI, FANCL, FANCM, MLH1, MSH2, MSH6, MUTYH, NF1, NF2, PALB2, PMS2, POLD1, POLE, PTCH1, PTEN, RAD51C, RAD51D, RET, SLX4, SMAD4, SMARCB1, SMARCE1, SPRED1, STK11, TP53* |
| 79 | *APC, BMPR1A, MLH1, MSH2, MSH6, MUTYH, PMS2, POLD1, POLE, PTEN, SMAD4, STK11* |
| 80 | *BRCA1, BRCA2, CDH1, CDK4, CDKN2A (p16), CDKN2A (p14), MLH1, MSH2, MSH6, PMS2, PTEN, STK11, TP53* |
| 81 | *ATM, BRCA1, BRCA2, CHEK2, PALB2, PTCH1, PTEN, STK11, TP53* |
| 82 | *APC, ATM, BMPR1A, BRCA1, BRCA2, CDH1, CDK4, CDKN2A, MLH1, MSH2, MSH6, MUTYH, PALB2, PMS2, POLD1, POLE, PTCH1, PTEN, SMAD4, STK11, TP53* |
| 83 | *APC, BMPR1A, CDH1, MLH1, MSH2, MSH6, MUTYH, PMS2, POLD1, POLE, PTEN, SMAD4, STK11, TP53* |
| 84 | *ATM, BRCA1, BRCA2, BRIP1, CDH1, CHEK2, MLH1, MSH2, MSH6, NF1, PALB2, PMS2, PTEN, RAD51C, RAD51D, STK11, TP53* |
| 85 | *APC, BMPR1A, MLH1, MSH2, MSH6, PMS2, SMAD4, STK11* |
| 86 | *APC, BMPR1A, BRCA1, BRCA2, CDH1, FH, FLCN, MET, MLH1, MSH2, MSH6, MUTYH, PMS2, PTEN, SDHB, SMAD4, STK11, TP53, VHL* |
| 87 | *APC, ATM, BMPR1A, BRCA1, BRCA2, CDK4, CDKN2A, CHEK2, HOXB13, MLH1, MSH2, MSH6, MUTYH, PMS2, PTEN, RET, SMAD4, STK11, TP53* |
| 88 | *APC, ATM, BMPR1A, BRCA1, BRCA2, CDH1, CHEK2, MLH1, MSH2, MSH6, MUTYH, PALB2, PMS2, PTEN, SMAD4, STK11, TP53* |
| 89 | *CDKN1B, MEN1, NF1, VHL* |
| 90 | *APC, BMPR1A, MLH1, MSH2, MSH6, MUTYH, PMS2, POLD1, POLE, SMAD4, STK11* |
| 91 | *APC, BMPR1A, MLH1, MSH2, MSH6, MUTYH, PMS2, POLD1, POLE, SMAD4, STK11* |
| 92 | *APC, ATM, BMPR1A, BRCA1, BRCA2, CDH1, MLH1, MSH2, MSH6, MUTYH, PALB2, PMS2, PTEN, SMAD4, STK11, TP53* |
| 93 | *APC, ATM, BMPR1A, BRCA1, BRCA2, CHEK2, HOXB13, MLH1, MSH2, MSH6, MUTYH, PALB2, PMS2, POLD1, POLE, PTEN, SMAD4, STK11, TP53* |
| 94 | *ATM, BRCA1, BRCA2, CDH1, HOXB13, MLH1, MSH2, MSH6, PALB2, PMS2, PTEN, STK11, TP53* |
| 95 | *APC, BMPR1A, BRCA1, BRCA2, CHEK2, MLH1, MSH2, MSH6, MUTYH, PALB2, PMS2, POLD1, POLE, PTEN, SMAD4, STK11, TP53* |
| 96 | *APC, ATM, BMPR1A, BRCA1, BRCA2, MLH1, MSH2, MSH6, MUTYH, PMS2, POLD1, POLE, PTEN, SMAD4, STK11, TP53* |
| 97 | *MUTYH* |
| 98 | *ATM, BARD1, BRCA1, BRCA2, BRIP1, CDH1, CHEK2, MLH1, MSH2, MSH6, NF1, PALB2, PMS2, PTEN, RAD51C, RAD51D, RB1, STK11, TP53* |
| 99 | *ATM, BRCA1, BRCA2, BRIP1, CHEK2, HOXB13, MLH1, MSH2, MSH6, MUTYH, PALB2, PMS2, PTCH1, RAD51C & TP53* |
| 100 | *APC, ATM, BMPR1A, BRCA1, BRCA2, CDH1, CHEK2, MLH1, MSH2, MSH6, MUTYH, PALB2, PMS2, POLD1, POLE, PTEN, SMAD4, STK11, TP53* |
| 101 | *APC, ATM, BMPR1A, BRCA1, BRCA2, CDK4, CDKN2A, CHEK2, MLH1, MSH2, MSH6, MUTYH, PALB2, PMS2, POLD1, POLE, PTCH1, PTEN, SMAD4, STK11, TP53* |
| 102 | *APC, BMPR1A, BRCA1, BRCA2, BRIP1, HOXB13, MLH1, MSH2, MSH6, MUTYH, PALB2, PMS2, PTEN, RAD51C, RAD51D, RET, SMAD4, STK11 & TP53* |
| 103 | *ATM, BRCA1, BRCA2, BRIP1, CHEK2, HOXB13, MLH1, MSH2, MSH6, PMS2, TP53* |
| 104 | *APC, ATM, BMPR1A, BRCA1, BRCA2, BRIP1, CHEK2, MLH1, MSH2, MSH6, MUTYH, PALB2, PMS2, POLD1, POLE, PTEN, RAD51C, RAD51D, SMAD4, STK11, TP53* |
| 105 | *APC, ATM, BMPR1A, BRCA1, BRCA2, CHEK2, MLH1, MSH2, MSH6, MUTYH, PALB2, PMS2, POLD1, POLE, PTEN, SMAD4, STK11, TP53* |
| 106 | *APC, BMPR1A, BRCA1, BRCA2, CDH1, CDK4, CDKN2A, CHEK2, MLH1, MSH2, MSH6, MUTYH, PMS2, POLD1, POLE, PTEN, SMAD4, STK11 & TP53* |
| 107 | *BRCA1, BRCA2, BRIP1, MLH1, MSH2, MSH6, PALB2, PMS2, PTEN, RAD51C, RAD51D, STK11, TP53* |
| 108 | *APC, ATM, BMPR1A, BRCA1, BRCA2, CHEK2, HOXB13, MLH1, MSH2, MSH6, MUTYH, PALB2, PMS2, POLD1, POLE, PTEN, SMAD4, STK11, TP53* |
| 109 | *APC, BMPR1A, BRCA2, CDK4, CDKN2A, CTRC, MLH1, MSH2, MSH6, MUTYH, PMS2, POLD1, POLE, PTEN, SMAD4, SPINK1, STK11, TP53* |
| 110 | *ATM, BRCA1, BRCA2, BRIP1, CHEK2, MLH1, MSH2, MSH6, PALB2, PMS2, PTEN, RAD51C, RAD51D, STK11, TP53* |
| 111 | *BRCA1, BRCA2, CDK4, CDKN2A, CHEK2, CTRC, MLH1, MSH2, MSH6, NF1, PALB2, PMS2, PTEN, SPINK1, STK11, TP53* |
| 112 | *APC, BMPR1A, CHEK2, MLH1, MSH2, MSH6, MUTYH, PMS2, POLD1, POLE, PTEN, RET, SMAD4, STK11* |
| 113 | *ATM, BRCA1, BRCA2, CHEK2, MLH1, MSH2, MSH6, NF1, PALB2, PMS2, PTEN, STK11, TP53* |
| 114 | *ATM, BRCA1, BRCA2, BRIP1, CHEK2, HOXB13, MLH1, MSH2, MSH6, NF1, PALB2, PMS2, PTCH1, PTEN, RAD51C, RAD51D, STK11, TP53* |
| 115 | *APC, ATM, BMPR1A, BRCA1, BRCA2, BRIP1, CDH1, CHEK2, ERCC4, FANCA, FANCB, FANCC, FANCD2, FANCE, FANCF, FANCG, FANCI, FANCL, FANCM, MLH1, MSH2, MSH6, MUTYH, NF1, PALB2, PMS2, POLD1, POLE, PTCH1, PTEN, SMAD4, STK11, TP53* |
| 116 | *BRCA1, BRCA2, FH, HOXB13, MLH1, MSH2, MSH6, NF1, PMS2, TP53* |
| 117 | *APC, BMPR1A, BRCA1, BRCA2, CHEK2, MLH1, MSH2, MSH6, MUTYH, PALB2, PMS2, POLD1, POLE, PTCH1, PTEN, SMAD4, STK11, TP53* |
| 118 | *ATM, BRCA1, BRCA2, BRIP1, CHEK2, MLH1, MSH2, MSH6, NF1, PALB2, PMS2, PTEN, RAD51C, RAD51D, STK11, TP53* |
| 119 | *BRCA1, BRCA2, CDK4, CDKN2A, CTRC, FH, HOXB13, MLH1, MSH2, MSH6, MUTYH, PALB2, PMS2, PTEN, SPINK1, STK11, TP53* |
| 120 | *RET* |
| 121 | *ATM, BRCA1, BRCA2, BRIP1, CDK4, CDKN2A, CHEK2, MLH1, MSH2, MSH6, PALB2, PMS2, PTEN, RAD51C, RAD51D, STK11, TP53* |
| 122 | *APC, ATM, BMPR1A, BRCA1, BRCA2, BRIP1, CDH1, CHEK2, HOXB13, MLH1, MSH2, MSH6, MUTYH, NF1, PALB2, PMS2, POLD1, POLE, PTEN, RAD51C, RAD51D, SMAD4, STK11, TP53* |
| 123 | *APC, ATM, BRCA1, BRCA2, CHEK2, MLH1, MSH2, MSH6, MUTYH, PALB2, PMS2, POLD1, POLE, PTEN, STK11, TP53* |
| 124 | *ATM, BRCA1, BRCA2, BRIP1, CDH1, CHEK2, MLH1, MSH2, MSH6, MUTYH, NF1, PALB2, PMS2, POLD1, POLE, PTEN, RAD51C, RAD51D, STK11, TP53* |
| 125 | *ATM, BRCA1, BRCA2, BRIP1, CDH1, CHEK2, MLH1, MSH2, MSH6, PALB2, PMS2, PTEN, RAD51C, RAD51D, STK11, TP53* |
| 126 | *ATM, BRCA1, BRCA2, CHEK2, NF1, PALB2, PTEN, STK11, TP53* |
| 127 | *ATM, BRCA1, BRCA2, BRIP1, CHEK2, FH, FLCN, MET, MLH1, MSH2, MSH6, NF1, PALB2, PMS2, PTCH1, PTEN, RAD51C, RAD51D, SDHB, STK11, TP53, VHL* |
| 128 | *APC, ATM, BMPR1A, BRCA1, BRCA2, CDH1, CHEK2, HOXB13, MLH1, MSH2, MSH6, MUTYH, PMS2, POLD1, POLE, PTEN, SMAD4, STK11, NBN* |
| 129 | *BAP1* |
| 130 | *ATM, BRCA1, BRCA2, BRIP1, CHEK2, MLH1, MSH2, MSH6, PALB2, PMS2, PTEN, RAD51C, RAD51D, STK11, TP53* |
| 131 | *ATM, BRCA1, BRCA2, CDH1, CDK4, CDKN2A, CHEK2, FH, FLCN, MET, MLH1, MSH2, MSH6, MUTYH, NF1, PALB2, PMS2, PTEN, SDHB, STK11, TP53, VHL* |
| 132 | *ATM, BAP1, BARD1, BRCA1, BRCA2, BRIP1, CDH1, ERCC4, FANCA, FANCB, FANCC, FANCD2, FANCE, FANCF, FANCG, FANCI, FANCL, FANCM, PALB2, PTEN, RAD51C, RAD51D, STK11, TP53* |
| 133 | *APC, ATM, BMPR1A, BRCA1, BRCA2, BRIP1, CDH1, CHEK2, MLH1, MSH2, MSH6, MUTYH, PALB2, PMS2, POLD1, POLE, PTEN, RAD51C, RAD51D, SMAD4, STK11, TP53* |
| 134 | *ATM, BMPR1A, BRCA1, BRCA2, CDH1, CDK4, CDKN2A, CHEK2, CTRC, MLH1, MSH2, MSH6, PALB2, PMS2, PTEN, SPINK1, STK11, TP53* |
| 135 | *APC, ATM, BMPR1A, BRCA1, BRCA2, BRIP1, CDH1, CHEK2, MLH1, MSH2, MSH6, MUTYH, NF1, NF2, PALB2, PMS2, PTCH1, PTEN, RAD51C, RAD51D, SMAD4, STK11, TP53* |
| 136 | *APC, ATM, BRCA1, BRCA2, BRIP1, CDH1, CHEK2, MLH1, MSH2, MSH6, PALB2, PMS2, PTEN, RAD51C, RAD51D, STK11, TP53* |
| 137 | *APC, BAP1, BMPR1A, CHEK2, FH, FLCN, MET, MLH1, MSH2, MSH6, MUTYH, PMS2, POLD1, POLE, PTEN, SDHB, SDHC, SDHD, SMAD4, STK11, TMEM127, VHL* |
| 138 | *ATM, BRCA1, BRCA2, BRIP1, CHEK2, MLH1, MSH2, MSH6, PALB2, PMS2, PTEN, RAD51C, RAD51D, STK11, TP53* |
| 139 | *APC, BMPR1A, BRCA1, BRCA2, CHEK2, MLH1, MSH2, MSH6, MUTYH, PMS2, POLD1, POLE, PTEN, SMAD4, STK11* |
| 140 | *APC, BMPR1A, BRCA1, BRCA2, BRIP1, CDH1, MLH1, MSH2, MSH6, MUTYH, PALB2, PMS2, POLD1, POLE, PTEN, RAD51C, RAD51D, SMAD4, STK11, TP53* |
| 141 | *APC, ATM, BMPR1A, BRCA1, BRCA2, CDKN2A, CHEK2, HOXB13, MLH1, MSH2, MSH6, MUTYH, PMS2, POLD1, POLE, PTEN, SMAD4, STK11, TP53* |
| 142 | *ATM, BRCA1, BRCA2, CDK4, CDKN2A, CHEK2, HOXB13, MLH1, MSH2, MSH6, PALB2, PMS2, PTEN, STK11, TP53* |
| 143 | *AIP, ATM, BAP1, BRCA1, BRCA2, CDH1, CHEK2, MLH1, MSH2, MSH6, PALB2, PMS2, PTCH1, PTEN, STK11, TP53* |
| 144 | *MSH6* |
| 145 | *ATM, BRCA1, BRCA2, CHEK2, HOXB13, MLH1, MSH2, MSH6, PALB2, PMS2, PTEN, STK11, TP53* |
| 146 | *APC, ATM, BMPR1A, BRCA1, BRCA2, CDH1, CHEK2, HOXB13, MLH1, MSH2, MSH6, MUTYH, PALB2, PMS2, POLD1, POLE, PTEN, SMAD4, STK11, TP53* |
| 147 | *APC, BMPR1A, MLH1, MSH2, MSH6, MUTYH, PMS2, POLD1, POLE, SMAD4, STK11* |
| 148 | *ATM, BRCA1, BRCA2, BRIP1, CDH1, CHEK2, MLH1, MSH2, MSH6, PALB2, PMS2, PTEN, RAD51C, RAD51D, RB1, STK11, TP53* |
| 149 | *APC, BMPR1A, BRCA1, BRCA2, CHEK2, MLH1, MSH2, MSH6, MUTYH, NF1, PALB2, PMS2, PTCH1, PTEN, RB1, SMAD4, STK11, TP53* |
| 150 | *APC, ATM, BMPR1A, BRCA1, BRCA2, CDK4, CDKN2A, CHEK2, HOXB13, MLH1, MSH2, MSH6, MUTYH, PMS2, POLD1, POLE, SMAD4, STK11, TP53* |
| 151 | *APC, ATM, BMPR1A, BRCA1, BRCA2, CDH1, CHEK2, MLH1, MSH2, MSH6, MUTYH, PALB2, PMS2, POLD1, POLE, PTEN, SMAD4, STK11, TP53* |
| 152 | *APC, ATM, BMPR1A, BRCA1, BRCA2, BRIP1, CDK4, CDKN2A, CHEK2, FH, HOXB13, MLH1, MSH2, MSH6, MUTYH, PALB2, PMS2, POLD1, POLE, PTCH1, PTEN, RAD51C, RAD51D, SMAD4, STK11, TP53* |
